# Development of Nanostructured Water Treatment Membranes Based on Thermotropic Liquid Crystals: Molecular Design of Sub‐Nanoporous Materials

**DOI:** 10.1002/advs.201700405

**Published:** 2017-12-18

**Authors:** Takeshi Sakamoto, Takafumi Ogawa, Hiroki Nada, Koji Nakatsuji, Masato Mitani, Bartolome Soberats, Ken Kawata, Masafumi Yoshio, Hiroki Tomioka, Takao Sasaki, Masahiro Kimura, Masahiro Henmi, Takashi Kato

**Affiliations:** ^1^ The University of Tokyo Hongo Bunkyo‐ku 113‐8656 Japan; ^2^ Toray Industries, Inc. Sonoyama Otsu Shiga 520‐0842 Japan; ^3^ National Institute of Advanced Industrial Science and Technology (AIST) Onogawa 16‐1 Tsukuba Ibaraki 305‐8569 Japan

**Keywords:** liquid crystals, membranes, polymers, self‐assembly, sub‐nanoporous materials

## Abstract

Supply of safe fresh water is currently one of the most important global issues. Membranes technologies are essential to treat water efficiently with low costs and energy consumption. Here, the development of self‐organized nanostructured water treatment membranes based on ionic liquid crystals composed of ammonium, imidazolium, and pyridinium moieties is reported. Membranes with preserved 1D or 3D self‐organized sub‐nanopores are obtained by photopolymerization of ionic columnar or bicontinuous cubic liquid crystals. These membranes show salt rejection ability, ion selectivity, and excellent water permeability. The relationships between the structures and the transport properties of water molecules and ionic solutes in the sub‐nanopores in the membranes are examined by molecular dynamics simulations. The results suggest that the volume of vacant space in the nanochannel greatly affects the water and ion permeability.

## 1 Introduction

An inadequate supply of safe fresh water is one of the most important global issues of our time.^1^ Water purification with polymer membranes is a promising method because high‐grade drinking water can be obtained with low energy consumption at a low cost.^1^, ^2^, ^3^, ^4^, ^5^, ^6^, ^7^ Current commercial membrane materials are cross‐linked polyamides and cellulose derivatives.^4^, ^5^, ^6^, ^7^ However, it is difficult to obtain ordered sub‐nanopores with uniform diameters in these conventional membranes. New materials such as carbon‐based materials,^8^, ^9^, ^10^ synthetic or recombinant channel peptides,^11^, ^12^, ^13^ polymerized liquid crystals,^14^, ^15^, ^16^, ^17^, ^18^, ^19^, ^20^ and block copolymers^21^, ^22^, ^23^, ^24^, ^25^ have been developed as components for water treatment membranes. These materials can be used to prepare membranes with ordered nanopores and sub‐nanopores. Self‐assembly of polymerizable liquid‐crystalline (LC) molecules^2^, ^14^, ^15^, ^16^, ^17^, ^18^, ^19^, ^20^, ^26^, ^27^, ^28^, ^29^, ^30^ and block copolymers^2^, ^21^, ^22^, ^23^, ^24^, ^25^ has attracted attention for the preparation of stable functional materials thin films because these materials offer uniformly sized nanopores and sub‐nanopores. In addition, the alignment of channels within these materials is expected to be easier than that for membranes consisting of carbon nanotubes. The structures and sizes of the self‐assembled channels are dependent on the molecular structures and intermolecular interactions. 1D, 2D, and 3D nanochannels are obtained from LC materials that exhibit columnar (Col), smectic (Sm), and bicontinuous cubic (Cub_bi_) phases, respectively.^26^, ^27^, ^28^, ^29^, ^30^ These LC nanoporous and sub‐nanoporous materials have been applied as water treatment membranes,^14^, ^15^, ^16^, ^17^, ^18^, ^19^, ^20^ ion conductors,^31^, ^32^, ^33^, ^34^, ^35^, ^36^, ^37^, ^38^ and organic molecular sieves.^39^, ^40^, ^41^, ^42^, ^43^ For block copolymers, self‐assembly of cylinder, lamellar, and gyroid structures leads to formation of 1D, 2D, and 3D nanoporous membranes, respectively.^21^, ^22^, ^23^, ^24^, ^25^ Pore diameters range from 0.5 to 2 nm for LC materials^15^, ^16^, ^17^, ^18^, ^19^, ^20^ and 5 to 50 nm for block copolymers.^21^, ^22^, ^23^, ^24^, ^25^ The sizes of the channels in LC materials are in range required for reverse osmosis (RO) and nanofiltration (NF) membranes stopping small molecules and ions, whereas porous films prepared from block copolymers can function as ultrafiltration (UF) or microfiltration membranes rejecting larger solutes_._ 1, 2, 3, 4

The ionic sub‐nanopores of thermotropic ionic LC materials have excellent potential for use as membranes for selective rejection of salts or hazardous substances from seawater or brackish water.^19^, ^20^ These membranes have sub‐nanopores with uniform diameters, which form by self‐organization of the liquid crystals. The moieties on the inside walls of the sub‐nanopores can be changed. Moreover, these membranes may have fewer defects than commercial membranes because LC fluid states of monomeric compounds are used before photopolymerization in the membrane preparation process. Lyotropic liquid crystals are of interest and were used in an earlier study to prepare polymer membranes with Cub_bi_ structures for water treatment.^14^, ^15^, ^16^, ^17^, ^18^

**Figure 1:**
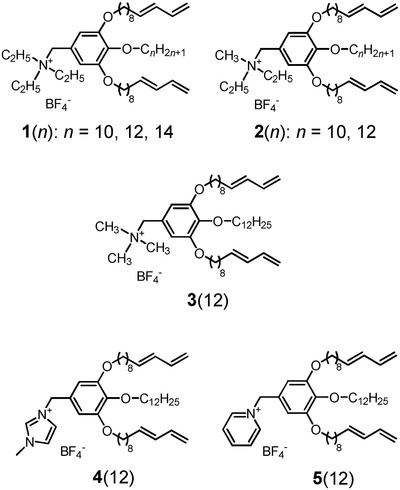
Molecular structures of the wedge‐shaped ionic molecules containing polymerizable diene moieties used in this study.

The aim of this study was to develop water purification materials based on nanostructured ionic polymer membranes with preserved thermotropic LC nanostructures. Wedge‐shaped ionic LC compounds containing hydrophobic polymerizable moieties (**1**(*n*)–**5**(*n*), **Figure** **1**) were designed and prepared. The water treatment properties of the nanostructured membranes with thermotropic hexagonal columnar (Col_h_) and Cub_bi_ LC structures were studied. Molecular dynamics (MD) simulations of water molecules and ionic solutes in the ionic nanochannels of the LC materials were examined to understand transport properties of water molecules and the rejection of ionic solutes in the sub‐nanoporous LC membranes.

## 2 Results and Discussion

### 2.1 Molecular Design and Thermotropic LC Properties of the Ionic Monomers

**Figure 2:**
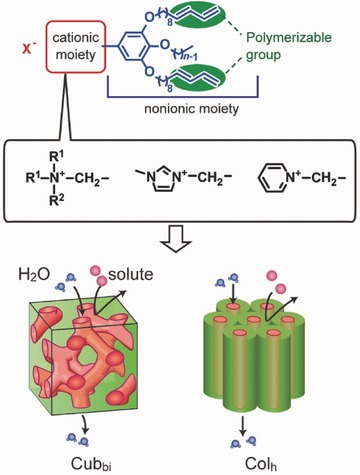
Molecular design in this work for development of a water treatment membrane based on thermotropic LC materials.

The principles of the molecular design for development of the thermotropic liquid crystals in this work are shown in **Figures** 1 and **2**. Molecules **1**(*n*)–**5**(*n*) were designed as ionic liquid crystals with ammonium, imidazolium, and pyridinium moieties. The cationic moieties for **1**(*n*), **2**(*n*), and **3**(*n*) are quaternary ammonium cations with various short alkyl chains. It has been reported that the size of an ionic moiety affects the assembled structure.^44^, ^45^ Compounds **4**(*n*) and **5**(*n*) contain *N*‐methylimidazolium and pyridinium moieties as their cationic groups, respectively. Compounds **1**(*n*)–**5**(*n*) also contain a tris(alkoxy)phenyl group with 1,3‐diene tails as polymerizable groups. These compounds were expected to exhibit thermotropic Col_h_ or Cub_bi_ phases with 1D or 3D uniform sub‐nanopores in the membranes.^44^, ^45^, ^46^, ^47^, ^48^ We previously reported on water treatment with self‐organized sub‐nanoporous membranes prepared by photopolymerization of the triethylammonium molecule **1**(12) in the Cub_bi_ phase (Figure 1).^19^ The Cub_bi_ membrane showed selective permeation for the sulfate ion in addition to a NaCl rejection rate and water permeability that were comparable to those of commercial NF membranes. Recently, we have achieved highly efficient virus rejection using nanoporous membranes based on Cub_bi_ LC compound **1**(14).^20^

The LC properties of compounds **1**(*n*)**–5**(*n*) are summarized in **Table** **1**. For compounds **1**(*n*) and **2**(*n*), the lengths of the alkyl chains on the 4‐position of the benzene ring was different. In our previous studies, compound **1**(12) was used to prepare an ion‐conducting film^36^ and a water treatment membrane.^19^ However, the LC isotropic transition temperature of **1**(12) is below room temperature. Compound **1**(14) was designed and obtained as a Cub_bi_ material with a higher LC isotropic transition temperature than that of **1**(12). A higher isotropic transition temperature makes preparation process of the membranes easier. Compound **1**(10) was not mesomorphic (Figure S1, Supporting Information). Compounds **2**(12)**–5**(12) formed Col_h_ phases (Figures S2–S11, Supporting Information). It is notable that only replacement of one ethyl group with a methyl group changed the mesophase of the wedge‐shaped compound from Cub_bi_ phase to Col phase.

**Table 1:** Thermal properties of ammonium compounds **1**(*n*)**−5**(*n*)

Compound	Ionic moiety	Phase transition behaviora)				
**1**(10)	Triethylammonium‐BF_4_	Cr	<−50			Isob)
**1**(12)c)	Triethylammonium‐BF_4_	Cr	−5 (4.3)	Cub_bi_	19 (0.07)	Iso
**1**(14)	Triethylammonium‐BF_4_	Cr	15 (2.4)	Cub_bi_	28 (0.49)	Iso
**2**(10)	Diethyl‐methylammonium‐BF_4_	Cr	−5 (19)	Col_h_	55 (0.59)	Iso
**2**(12)	Diethyl‐methylammonium‐BF_4_	Cr	8 (23.7)	Col_h_	62 (0.58)	Iso
**3**(12)d)	Trimethylammonium‐BF_4_	Cr	37	Col_h_	>120	Polym
**4**(12)d)	*N*‐Methylimidazolium‐BF_4_	Cr	42	Col_h_	>110	Polym
**5**(12)d)	Pyridinium‐BF_4_	Cr	40	Col_h_	>110	Polym

^a)^ Cr, crystalline; Cub_bi_, bicontinuous cubic; Col_h_, hexagonal columnar; Iso, isotropic; Polym, polymerization occurs in the LC phase. Transition temperatures (°C) and enthalpy changes (kJ mol^−1^, in parentheses) were determined by differential scanning calorimetry on the second heating cycle

^b)^ Compound **1**(10) did not show a phase transition above −50 °C

^c)^ Ref. 36

^d)^ Transition behavior in the first heating cycle.

For the compounds **1**(12), **2**(12), and **3**(12), those with smaller alkylammonium moieties showed higher isotropization temperatures. The higher isotropization temperatures of these compounds were partly attributed to stronger electrostatic interactions between the smaller cationic moiety and the anion in the ionic domain compared with compounds with a larger cationic moiety.^44^, ^45^ The LC phases of compounds **4**(12) and **5**(12) were stable over 100 °C. Ionic and π–π interactions may contribute to thermal stabilization of the Col phases. These monomers spontaneously polymerized because of thermal effects at temperatures over 100 °C.

### 2.2 Water Treatment with the LC Membranes

**Figure 3:**
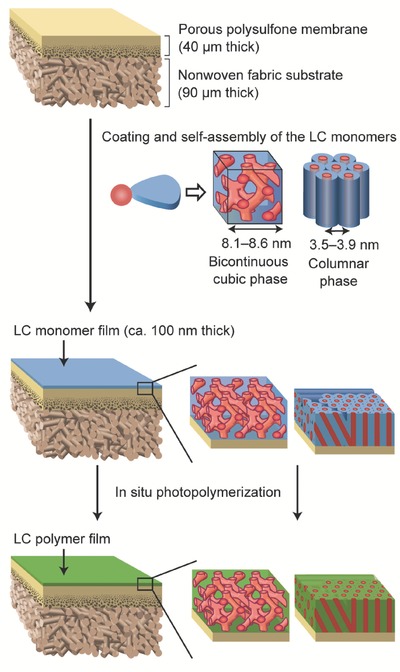
Schematic illustration for the preparation of the composite membranes from LC monomers.

Composite membranes for water treatment were prepared from the LC monomers **1**(*n*)–**5**(*n*) using the transcription method as previously reported (**Figure** **3**).^19^, ^20^ The LC polymer films were prepared by spin‐coating and subsequent photopolymerization, and were about 100 nm thick. The nanostructured LC polymer layer was supported by porous substrates of polysulfone and nonwoven polyester. The polysulfone and polyester layers were about 40 and 90 µm thick, respectively. These supporting layers can be used as ultrafiltration membranes having nanopores smaller than 10 nm^20^ and they give the composite membranes mechanical toughness.

**Figure 4:**
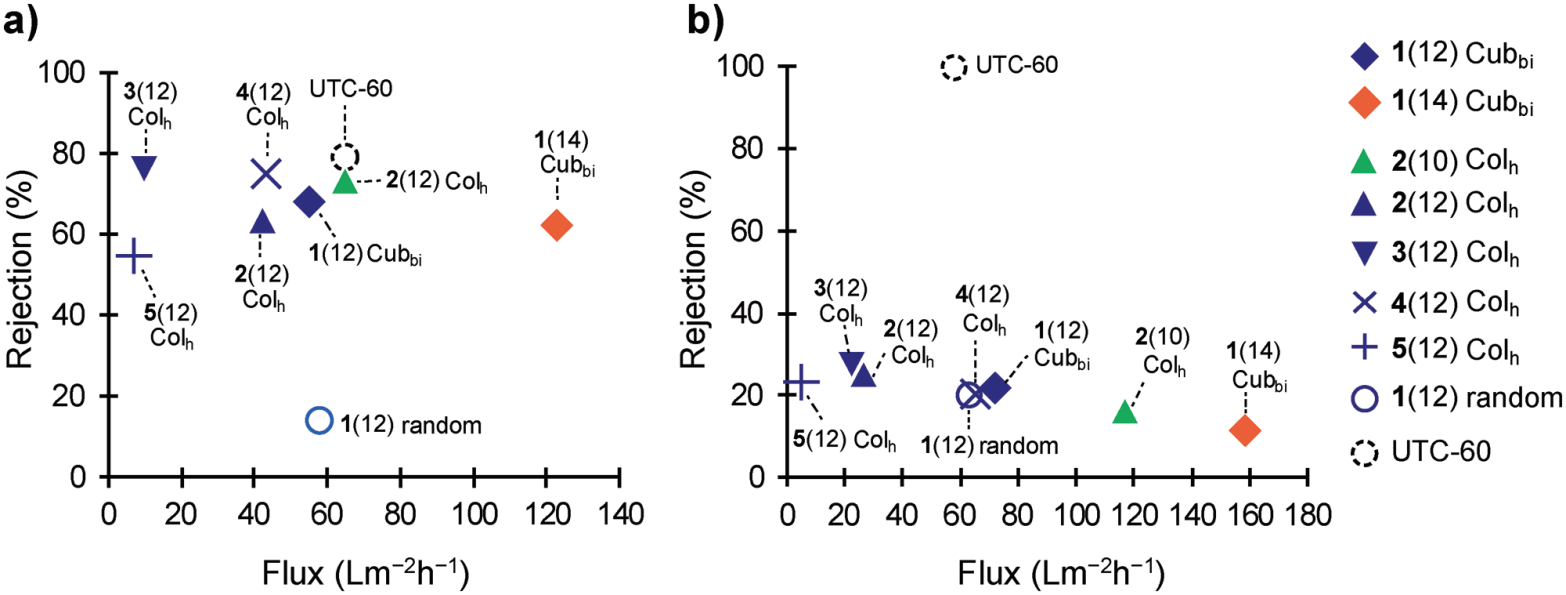
Rejection rates and fluxes through the composite membranes for aqueous solutions of a) NaCl and b) MgSO_4_. The maximum flux recorded for each membrane and rejection rate of the membrane are plotted. Conditions: operating pressure, 0.75 MPa; solution pH, 6.5; solution temperature, 25 °C; feeding rate, 3.5 L min^−1^; NaCl concentration, 500 ppm; MgSO_4_ concentration, 1500 ppm.

The water treatment properties of the composite membranes were examined using stainless steel cross‐flow filtration cells with an applied pressure of 0.75 MPa following established methods.^19^ The membranes were tested using aqueous solutions of NaCl and MgSO_4_ (**Table** **2**). The solution fluxes and rejection values for NaCl and MgSO_4_ with the membranes were measured, and **Figure** **4** shows the maximum flux recorded for each membrane. These membranes showed stable performance during the permeation test for tens of hours.

**Table 2:** NaCl and MgSO_4_ rejection with the LC composite membranes

Compound	State	NaCl rejection		MgSO_4_ rejection	
		Rejection [%]	Flux [L m^−2^ h^−1^]	Rejection [%]	Flux [L m^−2^ h^−1^]
**1**(12)	Cub_bi_	70 ± 6	39 ± 16	27 ± 5	59 ± 13
**1**(12)a)	Random	14 ± 9	58 ± 29	20 ± 4	63 ± 25
**1**(14)	Cub_bi_	69 ± 9	76 ± 47	16 ± 5	119 ± 40
**2**(10)	Col_h_	68 ± 10	39 ± 26	17 ± 5	86 ± 19
**2**(12)	Col_h_	62 ± 2	29 ± 13	26 ± 2	26
**3**(12)	Col_h_	73 ± 3	9 ± 1	30 ± 2	22
**4**(12)	Col_h_	65 ± 10	30 ± 13	24 ± 3	45 ± 20
**5**(12)	Col_h_	46 ± 9	5 ± 2	26 ± 3	8 ± 3
UTC‐60b)		79	65	99.6	58

Conditions: operating pressure, 0.75 MPa; solution pH, 6.5; solution temperature, 25 °C; feeding rate, 3.5 L min^−1^; NaCl concentration, 500 ppm; MgSO_4_ concentration, 1500 ppm.

^a)^ Ref. 19

^b)^ Refs. 49 and 50.

The membranes prepared from Cub_bi_ compound **1**(14) and Col_h_ compounds **2**(10), **2**(12), **3**(12), and **4**(12) exhibited over 60% rejection of NaCl, and their results were comparable to that of Cub_bi_ **1**(12). These membranes are the first examples of Col membranes based on thermotropic liquid crystals that exhibit the same level of NaCl rejection with commercial NF membrane. Compared with these membranes, the Col_h_ membrane prepared from compound **5**(12) had a lower NaCl rejection rate.

The rejection rates for MgSO_4_ with the LC **1**(14) and **2**(*n*)–**5**(12) membranes were lower than those of NaCl with the same membranes (Figure 4b). This behavior was similar to that of the Cub_bi_ **1**(12) membrane, which shows selective permeation of sulfate salts.^19^ However, this is an interesting result because polymer NF membranes usually reject sulfonate salts more efficiently than chloride salts, as observed with the commercial NF membrane UTC‐60^50^ and for a membrane prepared from **1**(12) in the isotropic state.^19^ This can be attributed to the larger hydrated radius of SO_4_^2−^ than that of Cl^−^. These results indicate that water molecules and solutes selectively permeate through the LC sub‐nanopores of the membrane. The salt rejection properties of the LC membranes prepared with quaternary ammonium, imidazolium, and pyridinium moieties exhibited similar trends. The stronger affinity between sulfate ions and the cation moieties in the LC sub‐nanopores^51^ likely causes the efficient capture and transport of sulfate salts and repulsion of the salts by the LC pores may be weaken.

The structures of the LC monomers also greatly affected the water permeability performances of the membranes. Notably, the maximum flux through the Cub_bi_ **1**(14) membrane during water treatment for NaCl rejection was above 120 L m^−2^ h^−1^ (Figure 4a). This value is approximately twice that through the Cub_bi_ **1**(12) membrane.^19^ We suppose that the membranes of polymerized Cub_bi_ **1**(14) formed more ordered and defectless structures than Cub_bi_ **1**(12). The decrease of defect and interruption in the sub‐nanopores might induce higher water flux. The fluxes through the Col_h_ membranes prepared from diethylmethylammonium compounds **2**(12) and **2**(10), and imidazolium compound **4**(12) were comparable to that through the commercial UTC‐60 membrane (Figure 4a). By contrast, the Col_h_ membranes prepared from trimethylammonium compound **3**(12) and pyridinium compound **5**(12) showed lower solution permeability than the Col_h_ **2**(12) membrane. The NaCl rejection with the Col_h_ **3**(12) membrane was similar to that of the Col_h_ **2**(*n*) membrane, and NaCl rejection with the **5**(12) membrane was lower than for these two membranes. These results suggest that the repulsion against NaCl and the affinity with water molecules of the Col_h_ **5**(12) membrane are weaker than those of the other LC materials used in the present study. The delocalized charge of pyridinium rings in the sub‐nanopores may cause this lower repulsion against NaCl. The differences of the water permeability of the LC membranes indicated that the water permeation through inside of the ionic pores overwhelmed the permeation through the hydrophobic part.

The rejection properties of nonionic solutes through the LC membranes were measured to examine the sizes of the sub‐nanopores (**Table** **3**). The rejection rate of glucose (monosaccharide) with the Cub_bi_ and Col LC membranes was less than 40%, although the molecular radius of glucose (0.37 nm)^52^, ^53^ is larger than the hydrated radius of Cl^−^ (0.33 nm) or Na^+^ (0.36 nm). The rejection rates of sucrose (disaccharide) and raffinose (trisaccharide), which have larger radii than glucose, were higher than that of glucose but lower than that of NaCl. These results indicated that the rejection of ionic species by the LC membranes was a result of interaction between the ionic sub‐nanopores and solute, and the sizes of the ions compared with the vacant space in the sub‐nanopores. This comparison among saccharides also revealed that the permeation/rejection of nonionic solutes by the LC membranes was dependent on the ionic moieties on the LC molecules. The ionic sub‐nanopores of **3**(12), which consist of smaller ammonium moieties, showed higher rejection ability than those of **1**(12) and **2**(12). These results indicate that the volume of vacant space in the ionic nanochannels of **3**(12) is smaller than that in other materials.

**Table 3:** Rejection of nonionic solutes and NaCl by the LC composite membranes

Solutea)	Stokes radius^52^, ^53^ [nm]	Rejection rates with the LC membranes [%]					
		**1**(12)	**1**(14)	**2**(10)	**2**(12)	**3**(12)	**4**(12)
Glucose	0.37	17	7	12	18	34	19
Sucrose	0.47	19	11	22	34	57	33
Raffinose	0.58	30	20	33	43	72	54
NaCl	Na^+^ (aq.): 0.36	67	68	71	72	74	65
	Cl^−^ (aq.): 0.33						

^a)^ Glucose, sucrose, and raffinose were all used at 1000 ppm.

The efficient permeation of water and the higher rejection rate of NaCl than MgSO_4_ with the Col_h_ membranes, such as the Col_h_ **2**(10) membrane, indicates that a large fraction of ionic sub‐nanopores in the Col_h_ structures fully span the membrane and water is mainly transported through the sub‐nanopores. Formation of sub‐nanopores from Col phases without any specific processes required for their alignment control is important for preparation of separation membranes that can effectively transport molecules and ions. There is a wide range of possible molecular designs for Col LC structures, whereas the range for Cub_bi_ LC structures is rather limited.^45^ This wider range for Col LC materials could be used to prepare membranes that exhibit highly selective separation, which is currently an important target. It is likely that the thinness of the LC films (≈100 nm) enabled more efficient transport of water molecules than a thicker membrane would. Thinner Col film provides shorter columnar sub‐nanopores between their surfaces than thicker Col films, which may decrease the possibility of the nanopores being discontinuous. It has been reported that Col channels in thinner films more frequently spontaneously align vertically than in thicker films.^54^, ^55^

The solution flux for the membranes is inversely proportional to the length of the pores and proportional to the number of pores. The path length for water molecules in the sub‐nanopores of the Cub_bi_ structures is about twice that in the vertically aligned straight pores of the Col membranes. In addition, rough estimation based on X‐ray diffraction (XRD) measurements suggests that the number of ionic liquid crystalline pores in the Col_h_ membranes would be similar to or slightly larger than in the Cub_bi_ membranes (Table S1, Supporting Information). These results indicate that a higher solution flux though the Col membranes could be obtained if homeotropical alignment in the Col structure increased.

### 2.3 Structural Analysis of the LC Nanopores and Molecular Dynamics Simulation in the Nanopores

The assembled structures of the LC molecules were examined by XRD measurements and a MD simulation. The XRD measurements revealed that the lattice parameters of the Cub_bi_ of compounds **1**(12) and **1**(14) were 8.1 and 8.6 nm, respectively. For the Col_h_ phases of **2**(10)**–5**(12), the intercolumnar distance were calculated as 3.5–3.9 nm from the XRD measurements (Figures S3–S11, Supporting Information). The self‐assembled structures of the LC monomers were preserved during the photopolymerization.

For the Col phases of compounds **2**(12)–**5**(12), there were about four wedge‐shaped molecules per 0.45‐nm‐thick slice of each nanochannel (Table S1, Supporting Information). For the Cub_bi_ structures, the estimation of the number of molecules forming the nanopores was less accurate than that for the Col structures because of junctions of their 3D interconnected structures.^56^ However, the 0.45‐nm‐thick slices of the Cub_bi_ lattices of **1**(12) and **1**(14) were composed of 22 and 24 molecules, respectively (Table S1, Supporting Information), and these layers contained four nanochannels and/or junctions. We assumed that the assembled structures that formed nanopores in the Cub_bi_ phases were similar to those in the Col_h_ phases.

The local structural features of the ionic sub‐nanopores and effects of the pore sizes on molecular or ion transport through the pores were examined by MD simulations.^57^ We assumed that the computer simulation revealed the behavior of the molecules and ions in the sub‐nanopores in the LC membranes. MD simulations have been used for water permeation through polyamide RO membranes,^58^, ^59^, ^60^, ^61^, ^62^, ^63^, ^64^, ^65^ carbon nanotubes,^66^, ^67^, ^68^, ^69^ and zeolite.^70^ However, to the best of our knowledge, this is the first example of the use of MD simulations for the analysis of the transport of water molecules and ions in LC polymer membranes with sub‐nanopores of uniform sizes and structures.

In this simulation, we focused on the stable structure of the assembled ammonium moieties forming the ionic nanopores and their effects on the transport properties of water molecules and ionic solutes. Triethylammonium (compounds **1**(*n*)), diethymethylammonium (compounds **2**(*n*)), and trimethylammonium (compound **3**(*n*)) moieties were examined for the LC monomers (Figure S12 and Table S2, Supporting Information). The alkoxy chains in the hydrophobic part of the LC monomers were eliminated and differences between **1**(12) and **1**(14) or **2**(10) and **2**(12) were not considered. The simulation method for stable structures of the ionic nanopores is described below and further details are given in the Supporting Information (Figure S13, Supporting Information). First, we supposed an LC tetramer layer was composed of four monomers, which were arranged radially in positions rotated by 90° so that their ammonium moieties were oriented toward the center of the layer. For simplicity, Cl^−^ was applied as an anion instead of BF_4_^−^. Next, four of the LC tetramers were stacked in the direction normal to the layer (the *z*‐axis direction) to construct an ionic nanopore. In the assembly, the second and forth layers were rotated by 45° around the center to form a close‐packed structure. The distance between the layers was fixed at 0.45 nm, which corresponds to the alkyl chain spacing. Then, several assemblies with different pore sizes were prepared by changing the distance, *R*, between the centers of the tetramer layer and the benzene ring of each LC monomer. Finally, an MD simulation for the energy minimization was performed for each assembly with 16 anions to obtain the stable structure of the nanopore at each *R*. During the simulation, the benzene rings of the LC monomers were fixed at their initial positions.

**Figure 5:**
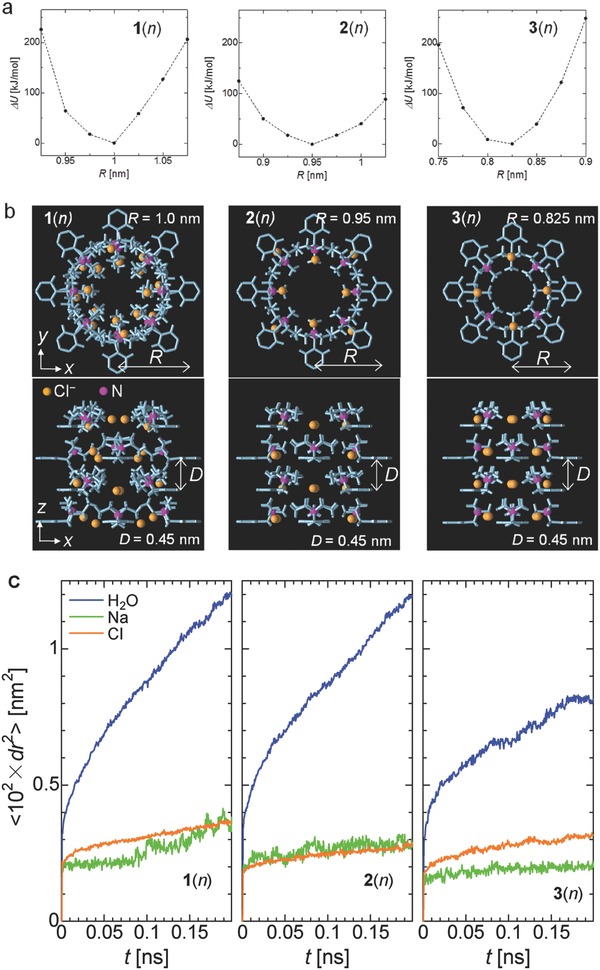
Simulation of ionic nanopores. a) Potential energy (*U*) for the stable structure of the assembly as a function of the distance between the centers of the tetramer layer and the benzene ring (*R*) for **1**(*n*), **2**(*n*), and **3**(*n*). The vertical axis represents the potential energy difference ∆*U* = *U* − *U*_min_, which is the minimum *U* estimated for each compound (*U* at *R* = 1.0 nm for **1**(*n*), *R* = 0.95 nm for **2**(*n*), and *R* = 0.825 nm for **3**(*n*)). b) The stable structures of the assemblies for **1**(*n*)–**3**(*n*). The alkyl chains, and H atoms of the LC monomers are not shown. c) The root‐mean‐square displacement, 〈d*r* ^2^〉, as a function of time, *t*, for water molecules, Na^+^ ions, and Cl^−^ ions in the ionic sub‐nanopores of **1**(*n*)–**3**(*n*).

**Figure** **5**a shows the potential energy, *U*, for the stable structure of the LC assembly as a function of *R* for **1**(*n*)–**3**(*n*). The minimum *U* occurred at *R* = 1.0 nm for **1**(*n*), *R* = 0.95 nm for **2**(*n*), and *R* = 0.825 nm for **3**(*n*). Figure 5b shows the stable structures of the assemblies that provided the minimum energy structures. These results suggest that the centers of the ionic nanopores of compounds **1**(*n*) and **2**(*n*) have more vacant space than in compound **3**(*n*).

For each of **1**(*n*)–**3**(*n*), using the minimum energy structure of the assembly, an MD simulation was performed to examine the movement of water molecules and ions in the ionic sub‐nanopores (for details see Section S5, Supporting Information). The MD simulation was performed for a system in which two copies of the assembly were stacked on the *z*‐axis so that the length of the channel was twice that of a single assembly. Water molecules, Na^+^ ions, and Cl^−^ ions were put into the sub‐nanopore. The number of water molecules in the pore was determined using a grand canonical Monte Carlo (GCMC) simulation. In the GCMC simulation, the chemical potential was set by assuming that a pressure of 1 atm was applied to the system. The mobilities of the water molecules and ions in the sub‐nanopores were examined by analyzing the root‐mean‐square displacement, 〈d*r* ^2^〉. The temperature was maintained at 298 K during the MD simulation.

Figure 5c shows the 〈d*r* ^2^〉 as a function of time, *t* for water molecules, Na^+^ ions, and Cl^−^ ions. The increase in 〈d*r* ^2^〉 with increasing *t* indicated that self‐diffusion occurred, and the slope of the 〈d*r* ^2^〉 function was proportional to the self‐diffusion coefficient. The larger the self‐diffusion coefficient of a species means the higher the mobility of the species. From the results (Figure 5c), it was obvious that the water molecules were more mobile than Na^+^ and Cl^−^ ions in the ionic sub‐nanopores of **1**(*n*)–**3**(*n*). Notably, the mobilities of water molecules and the ions were lower for **3**(*n*) than for **1**(*n*) and **2**(*n*). These results are consistent with the NaCl rejection rates for the LC membranes prepared from **1**(12)**–3**(12), and the lower water permeability of the Col_h_ **3**(12) membrane compared with the other LC membranes.

The different mobilities of ions and water molecules in the sub‐nanopores of compounds **1**(*n*), **2**(*n*), and **3**(*n*) could be caused by different volumes of vacant space in the pores and differences in the interactions between the solute ions and the ammonium moiety. In this simulation, the mobility of water molecules in the nanopores of compound **3**(*n*) increased if *R* of the nanopores was changed to 0.875 nm (Figure S14, Supporting Information). This result suggests that the different mobilities of water molecules for compounds **1**(*n*)–**3**(*n*) originated from the difference in the volume of vacant space in the nanopores. By contrast, the mobility of the ions did not change much even if *R* was changed to 0.875 nm. Thus, we speculate that the difference in the mobility of the ions for compounds **1**(*n*), **2**(*n*), and **3**(*n*) originated from differences in the interactions between the ions and the ammonium moiety.

## 3 Conclusion

To develop efficient water treatment membranes, wedge‐shaped ammonium, imidazolium, and pyridinium molecules containing polymerizable moieties were prepared. These organic ionic compounds form Cub_bi_ and Col_h_ phases depending on the structure of the cationic moiety and the alkyl chain length. The LC states of these compounds are successfully preserved by photopolymerization. These membranes function as sub‐nanoporous membranes for water treatment. Permeation tests using the composites membranes prepared from the LC monomers revealed that 3D interconnected sub‐nanopores of the Cub_bi_ structures and 1D sub‐nanopores of the Col_h_ phase span the membranes, and no procedures are required for control of their alignment. Membranes prepared from monomers with smaller quaternary ammonium moieties show lower permeability to water molecules, ions, and saccharides than membranes prepared with larger ammonium moieties. The assembled structures of the LC compounds were examined using MD simulations. The simulation results indicate that the lower permeability of nanopores composed of smaller ammonium moieties is mainly caused by the smaller volume of vacant space in the ionic pores compared with that in membranes prepared with larger ammonium moieties.

## 4 Experimental Section

*Materials and Methods*: Liquid‐crystalline properties were examined by an Olympus BH‐51 optical polarizing microscope equipped with a Mettler FP82HT hot‐stage or a Linkam LTS350 hot‐stage. Differential scanning calorimetry (DSC) measurements were conducted with a NETZCH DSC 204 Phoenix system at a cooling rate of 5 °C min^−1^. NMR spectra were recorded using a JEOL JNM‐ECX400 at 400 MHz for ^1^H NMR and at 100 MHz for ^13^C NMR in CDCl_3_. Chemical shifts of ^1^H and ^13^C NMR signals were quoted to internal standard Me_4_Si (δ = 0.00) and CDCl_3_ (δ = 77.00), respectively, and expressed by chemical shifts in ppm (δ), multiplicity, coupling constant (Hz), and relative intensity. Elemental analyses were carried out on a CE‐440 Elemental Analyser (Exeter Analytical Inc.). XRD patterns were obtained by a Rigaku RINT‐2500 diffractometer with a heating stage using a Ni‐filtered CuKα radiation. Matrix‐assisted laser desorption ionization time‐of‐flight mass spectra (MALDI‐TOF‐MS) were taken on a BRUKER autoflexTM speed spectrometer using dithranol as the matrix. UV irradiation was performed with a UV‐LED CCS HLDL100U system or a high‐pressure mercury lamp.

*Synthesis of LC Monomers and Preparation of Membranes*: Synthetic routes of the LC monomers and their details were described in Scheme S1 and the Supporting Information. All reagents of the highest quality were purchased from Aldrich, Kanto, Tokyo Kasei, or Wako, and were used as received.

The porous polysulfone layer and LC composite membranes were prepared using established methods.^19^, ^20^, ^71^ For compounds **1**(12)**, 1**(14)**,** and **2**(10), the LC monomer on the polymer support was heated at a temperature 20 °C higher than the isotropization temperature of each compounds, and then cooled to 10 °C. After cooling, UV light was irradiated to the LC monomer film on a polymer substrate using high‐pressure mercury lamp or the CCS LED lamp at 10 °C for 10 min. For compounds **2**(12) and **3**(12)–**5**(12), the composite was heated at 80 °C, and then cooled to around room temperature. After cooling, UV light was irradiated at room temperature for 10 min.

*Performance Test*: Rejection/permeation performances of the composite membranes were tested as follows:^19^, ^20^ Membrane discs of 7 cm in diameter were set into cross‐flow filtration cells of custom‐made equipment. An aqueous solution of each solute was supplied to the membranes under an operating pressure 0.75 MPa to perform membrane filtration. The temperature and the pH of the feed solution were controlled to be 25 °C and 6.5, respectively. The feed and permeate water were sampled after the operation continued for more than 3 h for stabilizing membrane performance. This procedure using each solute was executed one by one. The salt rejection rate was calculated based on the electrical conductivity of the feed and permeate water, which was measured using pH/EC METER WM‐50EG (DKK‐TOA Corp.). Rejection rate of nonionic solute consisting of a single component was calculated from the concentrations in the feed and permeate water measured by differential refractometer (SHIMADZU RID‐6A).

## Conflict of Interest

The authors declare no conflict of interest.

## Supporting information

Supplementary
