# Supplementary material for: Development of Nanostructured Water Treatment Membranes Based on Thermotropic Liquid Crystals: Molecular Design of Sub‐Nanoporous Materials
Source: Adv Sci (Weinh). 2017 Dec 18;5(1):1700405. doi: 10.1002/advs.201700405 (PMC5770667; doi:10.1002/advs.201700405)
Supplement: Supplementary file 1 — Supplementary [file ADVS-5-na-s001.pdf]

Copyright WILEY-VCH Verlag GmbH & Co. KGaA, 69469 Weinheim, Germany, 2017.

ADVANCED SCIENCE
Open Access

Supporting Information

for *Adv. Sci.,* DOI: 10.1002/advs.201700405

Development of Nanostructured Water Treatment Membranes Based on Thermotropic Liquid Crystals: Molecular Design of Sub-Nanoporous Materials

Takeshi Sakamoto, Takafumi Ogawa, Hiroki Nada,* Koji Nakatsuji, Masato Mitani, Bartolome Soberats, Ken Kawata, Masafumi Yoshio, Hiroki Tomioka, Takao Sasaki, Masahiro Kimura, Masahiro Henmi,* and Takashi Kato**

Copyright WILEY-VCH Verlag GmbH & Co. KGaA, 69469 Weinheim, Germany, 2016.

Supporting Information

Development of Nanostructured Water Treatment Membranes Based-on Thermotropic Liquid Crystals: Molecular Design of Sub-Nanoporous Materials

Takeshi Sakamoto, Takafumi Ogawa, Hiroki Nada,* Koji Nakatsuji, Masato Mitani, Bartolome Soberats, Ken Kawata, Masafumi Yoshio, Hiroki Tomioka, Takao Sasaki, Masahiro Kimura, Masahiro Henmi,* and Takashi Kato**

Table of Contents:

1. Synthesis of the Compounds

2. Liquid-Crystalline Properties

3. Simulation for the Ionic Channels

4. References for Supporting Information

1. Synthesis of the Compounds

Overall synthetic route of the monomers **1**(*n*)–**5**(*n*) were shown in **Scheme S1**. They were synthesized through similar method reported in our previous research.[1,2] Syntheses of **6**(14)–**9**(14), **1**(14)**-Cl**, **1**(14) were also reported in our recent paper in detail.[2]

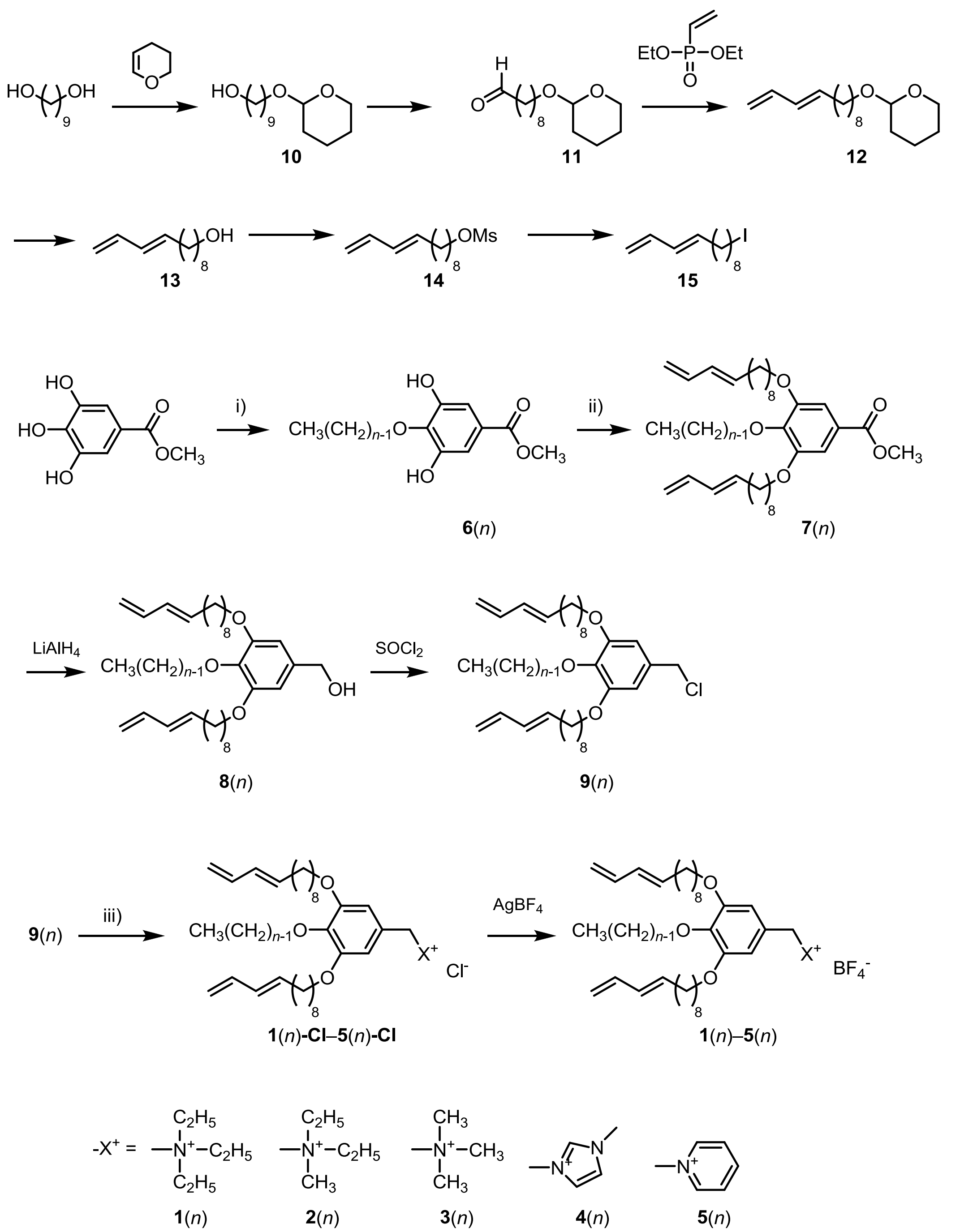

Scheme S1. Synthetic route of the liquid-crystalline compounds **1**(*n*)–**5**(*n*): i) NaH, $C_{12}H_{25}Br$ in DMF; ii) K_2CO_3, **15** in DMF; iii) triethylamine or diethylmethyamine or trimethylamine or *N*-methylimidazolium or pyridine.

9-(Tetrahydropyranyloxy)-1-nonanol (**10**)
9-(Tetrahydropyranyloxy)-1-nonanol (**10**) was synthesized according to references.[3] 1,9-nonandiol (50.1 g) was reacted with 3,4-dihydro-2*H*-pyran 27 mL in the biphasic systems of $NaHSO_4$ 12 g in biphasic solvent mixture of water (200 mL), hexane (500 mL), ethylacetate (70 mL), and dimethylsulfoxide (5 mL) for 16h at r.t. After removal of the water phase, the organic phase was washed with $NaHCO_3$ aq. for 5 times and dried over $MgSO_4$ filtered, and the solvent was removed in vacuo. The unreacted diol was removed with filtration using silica gel (eluent: hexane/ ethylacetate = 8:1) to give 9-(tetrahydropyranyloxy)-1-nonanol (59.98 g, 77.9 %) as colorless oil.
^{1}H NMR (400 MHz): δ = 4.58 (dd, *J* = 4.4, 2.4 Hz, 1H), 3.87 (ddd, *J* = 11.2, 7.6, 3.6 1H), 3.73 (dt, *J* = 9.6, 6.8 Hz, 1H), 3.65-3.58 (m, 2H), 3.52-3.46 (m, 1H), 3.37 (dt, *J* = 9.7, 6.8 Hz, 1H), 1.9–1.6 (m, 2H), 1.6–1.4 (m, 9H + water), 1.4–1.2 (m, 11H).

9-(Tetrahydropyranyloxy)-1-nonanal (**11**)
9-(Tetrahydropyranyloxy)-1-nonanal (**11**) was synthesized according to references.[4] Compound **10** (30.0 g, 123 mmol), 4-acetamido-2,2,6,6 tetramethylpiperidine 1-oxyl free radical (acetamido TEMPO, 0.50 g, 2.34mmol), tetrabutylammonium hydrogen sulfate (4.32 g,12.7mmol) were dissolved in dry CH_2Cl_2 (300 mL). K_2CO_3 (1.7g) was dispersed in the solution, and $NaClO{\cdot}5H_2O$ (28.05g, 170mmol) was added into the solution at 0°C over a period of 30 min. The reaction mixture was stirred at room temperature for 8 h. The crude product was obtained by washing with $Na_2S_2O_3$ aq., drying over Na_2SO_4, and concentration by using a rotary evaporator. The product was purified by flash column chromatography (silica gel, eluent: hexane/ethyl acetate = 9/1) to give compound **11** (22.92 g, 78%) as colorless oil.
^{1}H NMR (400 MHz): δ = 9.76 (t, *J* = 2.0 Hz, 1H), 4.57 (dd, *J* = 4.0, 2.8 Hz, 1H), 3.87 (ddd, *J* = 11.2, 7.6, 3.8 1H), 3.73 (dt, *J* = 9.6, 6.8 Hz, 1H), 3.52–3.46 (m, 1H), 3.38 (dt, *J* = 9.6, 6.6 Hz, 1H), 2.42 (td, *J* = 6.5, 1.7 Hz, 2H), 1.9–1.4 (m, 14H + water), 1.4–1.2 (m, 9H).

12-(Tetrahydropyranyloxy)-1,3-diene (**12**)[5]
Diethyl vinylphosphonate 18 mL (117 mmol) in dry THF 150 mL was cooled at -78 °C and 1.6 M *n*-BuLi in hexane 75 mL was added dropwisely and reacted for 30 min. After that, compound **11** (20.0 g, 82.5 mmol) and hexamethylphosphoric triamide (25 mL) were added into the solution and stirred at −78 °C for 3 h and at rt. for 3h. After quenching with NH_4Cl aq., the crude product was extracted with ethyl acetate, washed with water, dried over $MgSO_4$, and concentrated with a rotary evaporator. Colorless oily product **12** was obtained by flash column chromatography (silica gel, eluent: hexane/ethyl acetate = 9/1). (15.7g, 69.5 mmol, 84.7%)
^{1}H NMR (400 MHz): δ = 6.31 (ddd, *J* = 17.3, 10.4 Hz, 1H), 6.04 (dd, *J* = 15.0, 10.6 Hz, 1H), 5.70 (dt, *J* = 14.8, 7.3 Hz, 1H), 5.08 (d, *J* = 17.6 Hz, 1H), 4.94 (d, *J* = 9.2 Hz, 1H), 4.56 (dd, *J* = 4, 2.8 Hz, 1H), 3.87 (ddd, *J* = 11.2, 7.6, 3.8 Hz, 1H), 3.73 (dt, *J* = 9.6, 6.8 Hz, 1H), 3.52–3.46 (m, 1H), 3.38 (dt, *J* = 9.6, 6.8 Hz, 1H), 2.06 (q, *J* = 6.9Hz, 2H), 1.9–1.4 (m, 10H + water), 1.4–1.2 (m, 10H).

Dodeca-9,11-dien-1-ol (**13**)
A solution of compound **12** (8.68 g, 32.6 mmol) and *p*-TsOH (0.89 g) in MeOH (40 mL) containing H_2O (2 mL) was as stirred at r.t. After 2h of reaction, the solution was diluted with ethyl acetate and the organic phase was washed with sat. $NaHCO_3$ aq., dried over $MgSO_4$, filtered, and the solvent was removed in vacuo. The crude product was purified by silica

filtration (eluent: hexane/ethyl acetate = 5/1) to give compound **13** as colorless oil (5.34 g, 29.3 mmol, 90%).
^{1}H NMR (400 MHz): δ = 6.31 (dt, *J* = 17.1, 10.4 Hz, 1H), 6.04 (dd, *J* = 15.6, 10.8 Hz, 1H) , 5.70 (dt, *J* = 14.9, 7.3 Hz, 1H), 5.08 (d, *J* = 17.0 Hz, 1H), 4.95 (d, *J* = 10.0 Hz, 1H), 3.64 (q, *J* = 6.3 Hz, 2H), 2.07 (q, *J* = 7.3 Hz, 2H), 1.60–1.50 (m, 2H + water), 1.42–1.22 (m, 10H), 1.18 (t, *J* = 5.4Hz, 1H).
^{13}C NMR (100 MHz): δ = 137.43, 135.64, 130.95, 114.67, 63.17, 32.87, 32.60, 29.51, 29.43, 29.23, 29.19, 25.79.

Dodeca-9,11-dienyl-methanesulfate (**14**)
To a solution of compound **13** (5.33 g, 29.2 mmol) and triethylamine (5.23 g, 51.7 mmol) in dry CH_2Cl_2 (100 mL), methanesulfonyl chloride (5.08 g, 44.3 mmol) was added in ice bath and reacted at r.t. After 2h of reaction, the solution was diluted with chloroform and poured into water. The organic phase was separated, and washed with sat. $NaHCO_3$ aq. and sat. NH_4Cl aq. twice, dried over $MgSO_4$, filtered, and the solvent was removed in vacuo. The crude product was purified by silica filtration (eluent: hexane/ethyl acetate = 10/1) to give compound **14** as slightly colored oil. (6.76 g, 26.0 mmol, 88.8%).
^{1}H NMR (400 MHz): δ = 6.31 (dt, *J* = 16.8, 10.3 Hz, 1H), 6.03 (dd, *J* = 15.6, 10.8 Hz, 1H), 5.68 (dt, *J* = 14.9, 7.4 Hz, 1H), 5.08 (d, *J* = 17.2 Hz, 1H), 4.95 (d, *J* = 9.6 Hz, 1H), 4.22 (t, *J* = 6.8 Hz, 2H), 3.00 (s, 3 H), 2.07 (q, *J* = 7.1 Hz, 2H), 1.74 (dt, *J* = 14.7, 6.7 Hz, 2H), 1.44–1.22 (m, 10H).
^{13}C NMR (100 MHz): δ = 137.40, 135.51, 131.02, 114.74, 70.23, 37.46, 32.56, 29.32, 29.19, 29.17, 29.10, 29.03, 25.48.

12-Iodo-dodeca-1,3-diene (**15**)
Compound **14** (6.75 g, 25.9 mmol) and NaI (8.25 g, 55.0 mmol) in acetone (200 mL) were stirred for 3 h under reflux condition (80 °C). The mixture was poured into water and the organic phase extracted with hexane. The combined organic phases were concentrated in vacuo and purified by silica column (eluent: hexane) to give compound **15** as colorless oil (7.303 g, 25.2 mmol, 97.3%).
The obtained product was immediately used for next step.
^{1}H NMR (400 MHz): δ = 6.31 (dt, *J* = 17.2, 10.4 Hz, 1H), 6.04 (dd, *J* = 15.2, 10.4 Hz, 1H), 5.70 (dt, J = 14.9, 7.3 Hz, 1H), 5.08 (d, *J* = 16.4 Hz, 1H), 4.95 (d, *J* = 10.2 Hz, 1H), 3.19 (t, *J* = 7.1 Hz, 2H), 2.08 (q, *J* = 6.8 Hz, 2H), 1.82 (dt, *J* = 14.5, 7.2 Hz, 2H), 1.44-1.24 (m, 10 H).
^{13}C NMR (100 MHz): δ = 137.42, 135.52, 131.02, 114.72, 33.64, 32.60, 30.57, 29.34, 29.21, 29.15, 28.56, 7.34.

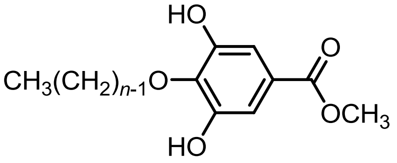

6(*n*)

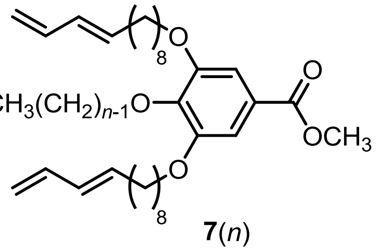

7(*n*)

Methyl-3,5-dihydroxy-4-dodecyloxybenzoate (**6**(12))
To a solution of methylgallate (10.37 g, 56.3 mmol) in DMF (130 mL), a suspension of NaH (2.11 g as an oil suspension, 52.8 mmol) in dry DMF (10 mL) were added in ice bath. After

1h stirring, 1-bromododecane (17.0 g, 68.27 mmol) was added to the solution and heated at 60°C for 3h. The mixture was diluted with ethyl acetate and poured into water. The organic phase was separated, and the aqueous phase was extracted three times with ethyl acetate. The combined organic phase was washed with sat. NH_4Cl aq. twice, dried over $MgSO_4$, filtered, and the solvent was removed in vacuo. The crude product was purified by flash column chromatography (silica gel, eluent: hexane/ethyl acetate = 4/1) and recrystallization from hexane solution (twice) to give compound **6**(12) as white powder (9.95 g, 28.2 mmol, 53.5 %).
^{1}H NMR (400 MHz): δ = 7.21 (s, 2H), 5.42-5.37 (br, 2H), 4.08 (t, *J* = 6.8 Hz, 2 H), 3.88 (s, 3 H), 1.79 (quin, *J* = 7.4 Hz, 2H), 1.50-1.20 (m, 18 H), 0.88 (t, *J* = 6.8 Hz, 3H).
^{13}C NMR (100 MHz): δ = 166.90, 148.86, 137.79, 125.91, 109.82, 74.14, 52.30, 31.97, 30.26, 29.68, 29.61, 29.58, 29.42, 29.39, 25.94, 22.74, 14.15.

Methyl-3,5-dihydroxy-4-decyloxybenzoate (**6**(10))
This compound was synthesized analogously to compound **6**(12) except using 1-bromodecane and purified by flash column chromatography (silica gel, eluent: hexane/ethyl acetate = 4/1) and recrystallization from hexane solution (twice) to give the desired product as white powder (4.73 g, 49.7% yield).
^{1}H NMR (400 MHz): δ = 7.21 (s, 2H), 5.39 (s, 2H), 4.08 (t, *J* = 6.8 Hz, 2 H), 3.88 (s, 3 H), 1.79 (quin, *J* = 7.3 Hz, 2H), 1.50-1.20 (m, 14H), 0.88 (t, *J* = 7.0 Hz, 3H).
^{13}C NMR (100 MHz): δ = 166.79, 148.83, 137.71, 126.01, 109.79, 74.20, 52.35, 31.96, 30.27, 29.54, 29.43, 29.37, 25.95, 22.76, 14.19.

Methyl-3,5-bis(9,11-dodecadienyl)oxy)-4-dodecyloxybenzoate (**7**(12))
A mixture of compound **6**(12) (3.78g, 10.7 mmol) and compound **15** (6.90 g, 23.6 mmol), and K_2CO_3 (15.0 g, 108.5 mmol) in dry DMF (40mL) was heated at 80 °C for 8 h under Ar. The organic phase was separated, and the aqueous phase was extracted three times with ethyl acetate. The combined organic phase was washed with sat. NH_4Cl aq., dried over $MgSO_4$, filtered, and the solvent was removed in vacuo. The crude product was purified by flash column chromatography (silica gel, eluent: hexane/ethyl acetate = 10/1) and recrystallization from EtOH solution to give compound **7**(12) as white solids (6.93 g, 10.2 mmol, 94.8 %).
^{1}H NMR (400 MHz): δ = 7.25 (s, 2H), 6.31 (dt, *J* = 17.3, 10.3 Hz, 2H), 6.04 (dd, *J* = 15.4, 10.6 Hz, 2H), 5.70 (dt, *J* = 14.9, 7.3 Hz, 2H), 5.08 (d, *J* = 17.6 Hz, 2H), 4.95 (d, *J* = 10.4 Hz, 2H), 4.05–3.95 (m, 6H), 3.88 (s, 3 H), 2.07 (q, *J* = 7.1 Hz, 4H), 1.85–1.70 (m, 6H), 1.50–1.20 (m, 38H), 0.88 (t, *J* = 6.6 Hz, 3H).
^{13}C NMR (100 MHz): δ= 167.01, 152.86, 142.35, 137.37, 135.42, 131.08, 124.75, 114.80, 108.00, 73.63, 69.13, 52.22, 32.57, 32.03, 30.41, 29.82, 29.76, 29.65, 29.49, 29.31, 29.22, 29.01, 26.15, 26.02, 22.80, 14.24.

Methyl-3,5-bis(9,11-dodecadienyl)oxy)-4-decyloxybenzoate (**7**(10)).
This compound was synthesized analogously to **7**(12) except using **6**(10) as the start compound and purified by flash column chromatography (silica gel, eluent: hexane/ethyl acetate = 10/1) and recrystallization from EtOH solution to give the desired product as white solids (6.75 g, 90.6% yield).
^{1}H NMR (400 MHz): δ = 7.25 (s, 2H), 6.30 (dt, *J* = 17.1, 10.4 Hz, 2H), 6.04 (dd, *J* = 15.6, 10.8 Hz, 2H), 5.69 (dt, *J* = 14.8, 7.3 Hz, 2H), 5.07 (d, *J* = 17.6 Hz, 2H), 4.94 (d, *J* = 10.0 Hz, 2H), 4.03–3.98 (m, 6H), 3.88 (s, 3 H), 2.08 (q, *J* = 6.8 Hz, 4H), 1.82–1.70 (m, 6H), 1.50–1.20 (m, 34H), 0.88 (t, *J* = 7.0 Hz, 3H).

^{13}C NMR (100 MHz): δ= 167.03, 152.89, 142.41, 137.43, 135.62, 130.96, 124.74, 114.68, 108.04, 73.56, 69.21, 52.20, 32.64, 32.03, 30.40, 29.82, 29.76, 29.65, 29.54, 29.49, 29.40, 29.36, 29.27, 26.14, 22.79, 14.22.
Elemental analysis calcd (%) for $C_{42}H_{68}O_5$: C, 77.25; H, 10.50. Found: C, 77.22; H, 10.82.
MS (MALDI-TOF): calcd. for $[M+H]^+$, 654.51 and $[M+Na]^+$, 675.50.. Found: 654.85, 675.75

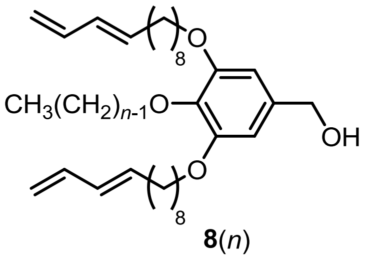

8(*n*)

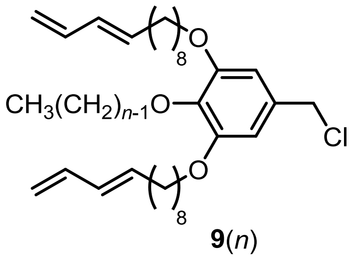

9(*n*)

3,5-Bis(9,11-dodecadienyl)oxy)-4-dodecyloxybenzyl alcohol (**8**(12)) was synthesized from compound **7**(12) (6.93 g, 10.2 mmol) according to the reference[1] and purified by flash column chromatography (silica gel, eluent: hexane/ethyl acetate = 4/1) to give compound **8**(12) as white solids (6.20 g, 9.49 mmol, 93.3% yield).
^{1}H NMR (400 MHz): δ = 6.54 (s, 2H), 6.30 (dt, *J* = 17.2, 10.3 Hz, 2H), 6.04 (dd, *J* = 15.4, 10.6 Hz, 2H), 5.70 (dt, *J* = 14.8, 7.3 Hz, 2H), 5.08 (d, *J* = 16.8 Hz, 2H), 4.95 (d, *J* = 10.4 Hz, 2H), 4.57 (d, *J* = 4.0 Hz, 2H), 4.00–3.92 (m, 6H), 2.08 (q, *J* = 6.9 Hz, 4H), 1.95–1.70 (m, 7H + water), 1.50–1.20 (m, 38H), 0.88 (t, *J* = 8.0 Hz, 3H).
^{13}C NMR (100 MHz): δ = 153.31, 137.56, 137.43, 136.21, 135.60, 130.98, 114.68, 105.38, 73.52, 69.13, 65.78, 32.64, 32.02, 30.42, 29.83, 29.77, 29.71, 29.56, 29.48, 29.42, 29.28, 26.23, 26.16, 22.79, 14.22.

3,5-Bis(9,11-dodecadienyl)oxy)-4-decyloxybenzyl alcohol (**8**(10)).
This compound was synthesized analogously to **8**(12) except using **7**(10) (6.48 g, 9.92 mmol) as the start compound and purified by flash column chromatography (silica gel, eluent: hexane/ethyl acetate = 4/1) to give the desired compound as white solids (5.39 g, 8.62 mmol, 86.8% yield).
^{1}H NMR (400 MHz): δ = 6.56 (s, 2H), 6.31 (dt, *J* = 17.2, 10.3 Hz, 2H), 6.05 (dd, *J* = 15.2, 10.0 Hz, 2H), 5.70 (dt, *J* = 14.9, 7.3 Hz, 2H), 5.08 (d, *J* = 17.2 Hz, 2H), 4.95 (d, *J* = 10.4 Hz, 2H), 4.59 (d, *J* = 5.6 Hz, 2H), 4.00-3.90 (m, 6H), 2.07 (q, *J* = 6.9 Hz, 4H), 1.82–1.65 (m, 6H), 1.50–1.20 (m, 35H + water), 0.88 (t, *J* = 6.6 Hz, 3H).
^{13}C NMR (100 MHz): δ = 153.28, 137.62, 137.34, 136.00, 135.53, 130.87, 114.58, 105.39, 73.42, 69.09, 65.68, 32.54, 31.94, 30.32, 29.74, 29.68, 29.61, 29.46, 29.40, 29.32, 29.18, 29.17, 26.13, 26.07, 22.70, 14.12.
Elemental analysis calcd (%) for $C_{41}H_{68}O_4$: C, 78.79; H, 10.97. Found: C, 79.12; H, 11.32.
MS (MALDI-TOF): calcd. for $[M+H]^+$, 625.51, $[M+Na]^+$, 647.50. Found: 626.08, 647.60

3,5-Bis(9,11-dodecadienyl)oxy)-4-dodecyloxybenzyl chloride (**9**(12)).
To a solution of compound **8**(12) (3.00 g, 4.59 mmol) in dry CH_2Cl_2 (50 ml) was added dropwise $SOCl_2$ (0.66 g, 5.55 mmol) with stirring at 0 °C. The mixture was stirred at room temperature for 3 h and slowly quenched with sat. $NaHCO_3$ aq. solution. The reaction mixture was extracted three times with $CHCl_3$ and washed with sat. NaCl aq., dried over $MgSO_4$,

filtered, and the solvent was removed in vacuo. The crude product was obtained as slightly red-colored solids (3.09 g) and immediately used for next step without additional purification.
^{1}H NMR (400 MHz) of δ = 6.56 (s, 2H), 6.31 (dt, *J* = 17.3, 10.3 Hz, 2H), 6.05 (dd, *J* = 15.6, 10.4 Hz, 2H), 5.70 (dt, *J* = 14.6, 7.2 Hz, 2H), 5.08 (d, *J* = 17.2 Hz, 2H), 4.95 (d, *J* = 10.8 Hz, 2H), 4.50 (s, 2H), 4.00-3.90 (m, 6H), 2.08 (q, *J* = 7.1 Hz, 4H), 1.82–1.65 (m, 6H), 1.50–1.20 (m, 38H), 0.89 (t, *J* = 6.6 Hz, 3H).

3,5-Bis(9,11-dodecadienyl)oxy)-4-decyloxybenzyl chloride (9(10)).
This compound was synthesized analogously to **9**(12) except using **8**(10) as the start compound the crude product was obtained as slightly red-colored solids.
^{1}H NMR (400 MHz): δ = 6.56 (s, 2H), 6.31 (dt, *J* = 17.1, 10.4 Hz, 2H), 6.05 (dd, *J* = 15.0, 10.4 Hz, 2H) , 5.70 (dt, *J* = 14.7, 7.0 Hz, 2H), 5.08 (d, *J* = 17.2 Hz, 2H), 4.95 (d, *J* = 10.0 Hz, 2H) , 4.51 (s, 2H), 4.00-3.92 (m, 6 H), 2.08 (q, *J* = 7.0 Hz, 4H), 1.82-1.70 (m, 6H), 1.47-1.26 (m, 42H), 0.88 (t, *J* = 6.8 Hz, 3H).

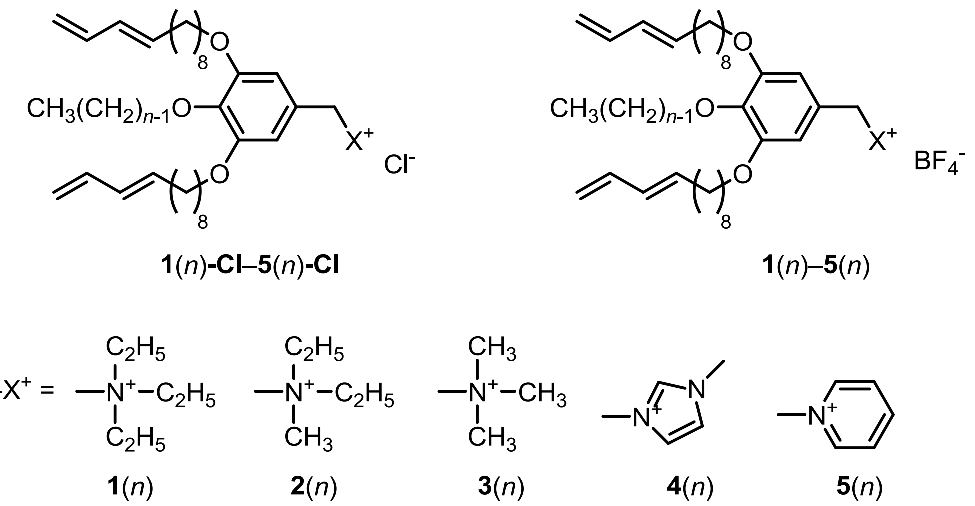

Triethyl-[3,5-bis(9,11-dodecadienyl)oxy)-4-dodecylbenzyl]ammnonium chloride (**1**(12)-**Cl**) was synthesized from compound **9**(12) (3.09 g, 4.60 mmol) according to the references[1] and purified through flash column chromatography (silica gel, eluent: CH_2Cl_2/methanol = 10/1) and recrystallization from acetone solution to give the product as white solids (1.12 g, 1.48 mmol, 32.1% yield).
^{1}H NMR (400 MHz): δ = 6.76 (s, 2 H), 6.31 (dt, *J* = 17.1, 10.4 Hz, 2H), 6.04 (dd, *J* = 15.0, 10.4 Hz, 2H), 5.70 (dt, *J* = 14.9, 7.3 Hz, 2H), 5.08 (d, *J* = 17.2 Hz, 2H), 4.95 (d, *J* = 11.6 Hz, 2H), 4.73 (s, 2 H), 4.01–3.94 (m, 6 H), 3.52–3.45 (m, 6H), 2.08 (q, *J* = 6.9 Hz, 4H), 1.82–1.70 (m, 6H), 1.47–1.26 (m, 47H), 0.88 (t, *J* = 6.8 Hz, 3H).

Triethyl-[3,5-bis(9,11-dodecadienyl)oxy)-4-dodecylbenzyl]ammnonium tetrafluoroborate (**1**(12)) was synthesized from compound **1**(12)-**Cl** (0.75 g, 97 mmol) according to the references[1] and purified through flash column chromatography (silica gel, eluent: CH_2Cl_2/methanol = 15/1) to give the product as transparent sticky solids (0.32 g, 39 mmmol, 40% yield).

^{1}H NMR (400 MHz): δ = 6.61 (s, 2 H), 6.30 (dt, *J* = 17.1, 10.4 Hz, 2H), 6.04 (dd, *J* = 15.4, 10.4 Hz, 2H), 5.70 (dt, *J* = 14.9, 7.3 Hz, 2H), 5.08 (d, *J* = 16.8 Hz, 2H), 4.95 (d, *J* = 10.4 Hz, 2H), 4.34 (s, 2 H), 3.98–3.92 (m, 6H), 3.29 (q, 7.3 Hz, 6 H), 2.07 (q, 7.1 Hz, 4H), 1.82–1.70 (m, 6H), 1.47–1.26 (m, 47H), 0.88 (t, *J* = 6.8 Hz, 3H).

Compound **1**(10)**-Cl** was synthesized analogously to **1**(12)**-Cl** except using **9**(10) (1.54 g, 2.40 mmol) as the start compound and purified through flash column chromatography (silica gel, eluent: CH_2Cl_2/methanol = 10/1) and recrystallization from acetone solution to give the product as sticky white solids (0.69 g, 0.93 mmol, 38%).
^{1}H NMR (400 MHz): δ = 6.76 (s, 2H), 6.31 (dt, *J* = 17.2, 10.3 Hz, 2H), 6.04 (dd, *J* = 15.4, 10.2 Hz, 2H) , 5.70 (dt, *J* = 14.9, 7.4 Hz, 2H), 5.08 (d, *J* = 17.2 Hz, 2H), 4.95 (d, *J* = 10.0 Hz, 2H) , 4.72 (s, 2H), 3.99–3.93 (m, 6 H), 3.56–3.32 (m, 6H), 2.08 (q, *J* = 7.1 Hz, 4H), 1.82–1.70 (m, 6H), 1.47-1.26 (m, 43H), 0.88 (t, *J* = 7.2 Hz, 3H).
^{13}C NMR (100 MHz): δ = 153.64, 140.40, 137.42, 135.60, 130.95, 121.83, 114.70, 111.42, 73.64, 69.79, 62.44, 53.14, 32.63, 32.02, 30.41, 29.82, 29.76, 29.66, 29.55, 29.48, 29.28, 26.19, 22.78, 14.21, 8.70.
Elemental analysis calcd (%) for $C_{47}H_{82}ClNO_3$: C, 75.81; H, 11.10; N, 1.88. Found: C, 74.51; H, 11.52; N, 2.04.
MS (MALDI-TOF): calcd. for $[M - Cl]^+$, 708.63. Found: 708.71.

Compound **1**(10) was synthesized analogously to **1**(12) except using **1**(10)-**Cl** (0.60 g, 0.81 mmmol) as the start compound and purified through flash column chromatography (silica gel, eluent: CH_2Cl_2/methanol = 15/1) to give the product as sticky colorless paste (0.26 g, 0.33 mmol, 41%).
^{1}H NMR (400 MHz): δ = 6.61 (s, 2H), 6.30 (dt, *J* = 17.1, 10.4 Hz, 2H), 6.04 (dd, *J* = 15.6, 10.4 Hz, 2H) , 5.70 (dt, *J* = 14.9, 7.3 Hz, 2H), 5.08 (d, *J* = 17.2 Hz, 2H), 4.95 (d, *J* = 11.2 Hz, 2H) , 4.34 (s, 2H), 3.98–3.91 (m, 6 H), 3.28 (q, 7.3 Hz, 6H), 2.07 (q, *J* = 7.1 Hz, 4H), 1.82–1.70 (m, 6H), 1.47–1.26 (m, 43H), 0.88 (t, *J* = 7.2 Hz, 3H).
^{13}C NMR (100 MHz): δ = 153.72, 140.37, 137.43, 135.62, 130.96, 121.34, 114.69, 110.91, 73.63, 69.60, 61.86, 52.82, 32.64, 32.03, 30.43, 29.83, 29.78, 29.67, 29.56, 29.48, 29.29, 26.17, 22.79, 14.22, 8.02.
Elemental analysis calcd (%) for $C_{47}H_{82}BF_4NO_3$: C, 70.92; H, 10.38; N, 1.76. Found: C, 70.77; H, 10.34; N, 1.97.
MS (MALDI-TOF): calcd. for $[M - BF_4]^+$, 708.63. Found: 708.73

Diethylmethyl-[3,5-bis(9,11-dodecadienyl)oxy)-4-dodecylbenzyl]ammnonium chloride (**2**(12)-**Cl**):
To a solution of compound **9**(12) (0.93 g, 1.39 mmmol), diethylmethyamine (3 mL) was added and heated at 70 °C for 24 h in a pressure tube. The reaction mixture was poured into water and extracted three times with $CHCl_3$. The organic phase was washed with 5% HCl aq. and sat. NaCl aq., dried over $MgSO_4$, filtered, and the solvent was removed in vacuo. The crude product was purified by flash column chromatography (silica gel, eluent: CH_2Cl_2/methanol = 10/1) and recrystallization in acetone to give compound **2**(12)-**Cl** (0.72 g, 0.95 mmol, 69%) as white solids.
^{1}H NMR (400 MHz): δ = 6.86 (s, 2 H), 6.31 (dt, *J* = 17.1, 10.4 Hz, 2H), 6.04 (dd, *J* = 15.6, 10.4 Hz, 2H) , 5.70 (dt, *J* = 15.1, 7.4 Hz, 2H), 5.08 (d, *J* = 16.8 Hz, 2H), 4.95 (d, *J* = 10.4 Hz, 2H), 4.84 (s, 2 H), 4.01–3.94 (m, 6H), 3.63-3.55 (m, 4H), 3.20 (s, 3H), 2.07 (q, *J* = 7.1 Hz, 4H), 1.82–1.70 (m, 6H), 1.47–1.22 (m, 44H), 0.88 (t, *J* = 6.8 Hz, 3H).

^{13}C NMR (100 MHz):δ = 153.54, 140.33, 137.42, 135.61, 130.95, 121.74, 114.70, 111.93, 73.58, 69.76, 65.25, 54.92, 46.76, 32.64, 32.02, 30.43, 29.83, 29.78, 29.68, 29.55, 29.47, 29.28, 26.20, 22.78, 14.21, 8.36.
Elemental analysis calcd (%) for $C_{48}H_{84}ClNO_3$: C, 75.99; H, 11.16; N, 1.85. Found: C, 73.49; H, 11.24; N, 2.01.
MS (MALDI-TOF): calcd. for $[M - Cl]^+$, 722.65. Found: 722.63.

Compound **2**(12) was synthesized analogously to **1**(12) except for using **2**(12)**-Cl** (0.70 g, 0.92 mmol) as the start compound and purified through flash column chromatography (silica gel, eluent: CH_2Cl_2/methanol = 15/1) to give the product as white solids (0.46 g, 0.57 mmol, 62%).
^{1}H NMR (400 MHz): δ = 6.67 (s, 2 H), 6.30 (dt, *J* = 17.2, 10.3 Hz, 2H), 6.04 (dd, *J* = 15.6, 10.4 Hz, 2H) , 5.69 (dt, *J* = 14.8, 7.3 Hz, 2H), 5.07 (d, *J* = 17.2 Hz, 2H), 4.94 (d, *J* = 10.0 Hz, 2H), 4.38 (s, 2 H), 3.99–3.92 (m, 6H), 3.40–3.22 (m, 4H), 2.94 (s, 3H), 2.07 (q, *J* = 7.1 Hz, 4H), 1.82-1.70 (m, 6H), 1.47–1.22 (m, 44H), 0.88 (t, *J* = 6.8 Hz, 3H).
^{13}C NMR (100 MHz): δ = 153.66, 140.32, 137.43, 135.62, 130.95, 121.18, 114.69, 111.39, 73.60, 69.60, 65.90, 55.36, 46.59, 32.65, 32.03, 30.44, 29.83, 29.79, 29.69, 29.56, 29.48, 29.29, 26.18, 22.78, 14.22, 8.12.
Elemental analysis calcd (%) for $C_{48}H_{84}BF_4NO_3$: C, 71.18; H, 10.45; N, 1.73. Found: C, 71.07; H, 10.63; N, 1.91
MS (MALDI-TOF): calcd. for $[M - BF_4]^+$, 722.65. Found: 722.88.

Compound **2**(10)**-Cl** was synthesized analogously to **2**(12)**-Cl** except using **9**(10) (2.13 g, 3.31 mmmol) as the start compound and purified through flash column chromatography (silica gel, eluent: CH_2Cl_2/methanol = 10/1) and recrystallization from acetone solution to give the product as white solids (1.24 g, 1.70 mmol, 51.4%).
^{1}H NMR (400 MHz): δ = 6.86 (s, 2 H), 6.30 (dt, *J* = 17.1, 10.4 Hz, 2H), 6.03 (dd, *J* = 15.6, 10.4 Hz, 2H) , 5.69 (dt, *J* = 14.8, 7.3 Hz, 2H), 5.03 (d, *J* = 16.8 Hz, 2H), 4.94 (d, *J* = 11.2 Hz, 2H), 4.87 (s, 2 H), 4.01-3.90 (m, 6H), 3.57 (q, *J* = 7.3 Hz, 4H), 3.19 (s, 3H), 2.07 (q, *J* = 7.1 Hz, 4H), 1.82-1.70 (m, 6H), 1.47-1.22 (m, 40H), 0.88 (t, *J* = 6.8 Hz, 3H).
^{13}C NMR (100 MHz): δ = 153.56, 140.31, 137.42, 135.61, 130.95, 121.43, 114.66, 111.31, 73.57, 69.51, 65.76, 61.11, 55.47, 46.50, 32.64, 32.02, 30.45, 29.83, 29.77, 29.68, 29.56, 29.49, 29.29, 26.19, 22.78, 14.21, 8.36.
Elemental analysis calcd (%) for $C_{46}H_{80}ClNO_3$: C, 75.62; H, 11.04; N, 1.92; O, 6.57. Found: C, 74.15; H, 11.01; N, 2.68.
MS (MALDI-TOF): calcd. for $[M - Cl]^+$, 694.61. Found 694.97

Compound **2**(10) was synthesized analogously to **1**(12) except for using **2**(10)**-Cl** (1.10g, 1.51 mmol) as the start compound and purified through flash column chromatography (silica gel, eluent: CH_2Cl_2/methanol = 15/1) to give the product as sticky colorless solids (1.06 g, 1.35 mmol, 90.0%).
^{1}H NMR (400 MHz): δ = 6.68 (s, 2 H), 6.30 (dt, *J* = 17.1, 10.5 Hz, 2H), 6.04 (dd, *J* = 15.6, 10.4 Hz, 2H) , 5.70 (dt, *J* = 14.8, 7.4 Hz, 2H), 5.03 (d, *J* = 17.2 Hz, 2H), 4.95 (d, *J* = 10.4 Hz, 2H), 4.35 (s, 2 H), 4.01-3.90 (m, 6H), 3.40-3.22 (m, 4H), 2.91 (s, 3H), 2.05 (q, *J* = 7.1 Hz, 4H), 1.82-1.70 (m, 6H), 1.47-1.22 (m, 40H), 0.88 (t, *J* = 6.8 Hz, 3H).
^{13}C NMR (100 MHz): δ = 153.59, 140.13, 137.43, 135.61, 130.95, 121.43, 114.66, 111.31, 73.57, 69.51, 65.76, 61.11, 55.47, 46.50, 32.64, 32.02, 30.45, 29.83, 29.77, 29.68, 29.56, 29.49, 29.29, 26.19, 22.78, 14.21, 8.04.

Elemental analysis calcd (%) for $C_{46}H_{80}BF_4NO_3$: C, 70.66; H, 10.31; N, 1.79. Found: C, 70.89; H, 10.38; N, 2.06
MS (MALDI-TOF): calcd. for $[M - BF_4]^+$, 694.61. Found 694.91

Trimethyl-[3,5-bis(9,11-dodecadienyl)oxy)-4-octylbenzyl]ammnonium chloride (**3**(12)-**Cl**):
To a solution of compound **9**(12) (1.11 g, 1.65 mmol) in acetonitrile (5 mL) was added trimethyamine in water (ca. 4.3 mol L^{-1}, 2 mL) and heated at 75 °C for 6 h. The reaction mixture was poured into water and extracted three times with $CHCl_3$. The organic phase was washed with 5% HCl aq. and sat. NaCl aq., dried over $MgSO_4$, filtered, and the solvent was removed in vacuo. The crude product was purified by flash column chromatography (silica gel, eluent: CH_2Cl_2/methanol = 10/1) and recrystallization in acetone to give **3**(12)-**Cl** (0.36 g, 0.50 mmol, 30 %) as white solids.
^{1}H NMR (400 MHz): δ = 6.77 (s, 2 H), 6.30 (dt, *J* = 16.8, 10.3 Hz, 2H), 6.04 (dd, *J* = 15.4, 10.6 Hz, 2H), 5.69 (dt, *J* = 15.1, 7.2 Hz, 2H), 5.08 (d, *J* = 17.2 Hz, 2H), 4.94 (d, *J* = 10.0 Hz, 2H), 4.92 (s, 2H), 3.99-3.92 (m, 6 H), 3.36 (s, 9H), 2.06 (q, 6.9 Hz, 4H), 1.82-1.70 (m, 6H), 1.47-1.26 (m, 38H), 0.88 (t, *J* = 7.2 Hz, 3H).
^{13}C NMR (100 MHz): δ = 153.61, 140.39, 137.42, 135.60, 130.96, 122.10, 114.69, 111.57, 73.61, 69.69, 63.01, 32.64, 32.02, 30.44, 29.83, 29.78, 29.68, 29.54, 29.47, 29.29, 26.20, 22.78, 14.21.
Elemental analysis calcd (%) for $C_{46}H_{80}ClNO_3$: C, 75.62; H, 11.04; N, 1.92. Found: C, 71.56; H, 10.65; N, 1.90.
MS (MALDI-TOF): calcd. for $[M - Cl]^+$, 694.61. Found: 694.94.

Compound **3**(12) was synthesized analogously to **1**(12) except for using **3**(12)-**Cl** (0.31 g, 0.42 mmol) as the start compound and purified through flash column chromatography (silica gel, eluent: CH_2Cl_2/methanol = 15/1) to give the product as white solids (0.25 g, 0.32 mmol, 76%).
^{1}H NMR (400 MHz): δ = 6.67 (s, 2 H), 6.27 (dt, *J* = 17.1, 10.3 Hz, 2H), 6.02 (dd, *J* = 15.1, 10.3 Hz, 2H) , 5.71 (dt,*J* = 15.1, 7.2 Hz, 2H), 5.07 (d, *J* = 17.1 Hz, 2H), 4.95 (d, *J* = 10.2 Hz, 2H) , 4.42 (s, 2 H), 3.96 (m, 6 H), 3.16 (s, 9 H), 2.08 (q, 6.9 Hz, 4H), 1.82–1.70 (m, 6H), 1.47–1.26 (m, 38 H), 0.86 (t, *J* = 6.8 Hz, 3H).
^{13}C NMR (100 MHz) : δ = 153.60, 137.32, 135.49, 130.86, 121.32, 114.57, 110.83, 73.52, 69.49, 52.72, 32.54, 31.92, 30.34, 29.74, 29.72, 29.68, 29.58,29.45, 29.37, 29.19, 29.17, 26.08, 22.68, 14.10, 7.89.
Elemental analysis calcd (%) for $C_{46}H_{80}BF_4NO_3$: C, 70.66; H, 10.31; N, 1.79. Found: C, 70.67; H, 10.76; N, 1.88.
MS (MALDI-TOF): calcd. for $[M - BF_4]^+$, 694.61. Found: 694.87.

1-Methyl-3-[3,5-bis(9,11-dodecadienyl)oxy)-4-dodecylbenzyl]imidazolium chloride (**4**(12)-**Cl**):
To a solution of compound **9**(12) (1.43 g, 2.14 mmol) in dry toluene (5 mL), 1-methylimidazole (3 mL) was added and heated at 70 °C for 40 h. The reaction mixture was poured into water and extracted three times with $CHCl_3$. The organic phase was washed with 5% HCl aq. and sat. NaCl aq., dried over $MgSO_4$, filtered, and the solvent was removed in vacuo. The crude product was purified by flash column chromatography (silica gel, eluent: CH_2Cl_2/methanol = 10/1) and recrystallization in acetone to give **4**(12)-**Cl** (0.80 g, 1.1 mmol, 50%) as white solids.

^{1}H NMR (400 MHz): δ = 11.04 (s, H), 7.13 (d, *J* = 16.8Hz, 2H), 6.64 (s, 2 H), 6.30 (dt, *J* = 17.2, 10.3 Hz, 2H), 6.04 (dd, *J* = 15.0, 10.6 Hz, 2H), 5.70 (dt,*J* = 14.8, 7.3 Hz, 2H), 5.42 (s, 2H), 5.08 (d, *J* = 17.2 Hz, 2H), 4.95 (d, *J* = 10.0 Hz, 2H), 4.08 (s, 3H), 3.97-3.89 (m, 6H), 2.06 (q, *J* = 7.1 Hz, 4H), 1.77–1.69 (m, 6H), 1.47–1.26 (m, 38 H), 0.88 (t, *J* = 6.8 Hz, 3H).
^{13}C NMR (100 MHz): δ = 153.94, 139.18, 138.88, 137.42, 135.60, 130.95, 127.51, 122.71, 121.22, 114.68, 107.76, 73.55, 69.56, 36.75, 32.63, 32.01, 30.40, 29.67, 29.55, 29.44, 29.28, 26.16, 22.77, 14.20
Elemental analysis calcd (%) for $C_{47}H_{77}ClN_2O_3$: C, 74.81; H, 10.10; N, 3.79. Found: C, 72.07; H, 9.72; N, 1.78.
MS (MALDI-TOF): calcd. for $[M - Cl]^+$, 717.59. Found 717.69.

Compound **4**(12) was synthesized analogously to **1**(12) except for using **4**(12)**-Cl** (0.70 g, 0.93 mmol) as the start compound and purified through flash column chromatography (silica gel, eluent: CH_2Cl_2/methanol = 15/1) to give the product as white solids (0.62 g, 0.77 mmol, 83%).
^{1}H NMR (400 MHz): δ = 8.92 (s, H), 7.15 (d, *J* = 8.8Hz, 2H), 6.59 (s, 2 H), 6.30 (dt, *J* = 17.3, 10.4 Hz, 2H), 6.04 (dd, *J* = 15.6, 10.4 Hz, 2H), 5.70 (dt,*J* = 15.2, 7.5 Hz, 2H), 5.20 (s, 2H), 5.06 (d, *J* = 17.2 Hz, 2H), 4.94 (d, *J* = 10.8 Hz, 2H) , 3.97–3.89 (m, 9H), 2.07 (q, *J* = 6.9Hz, 4H), 1.82–1.69 (m, 6H), 1.47–1.26 (m, 38 H), 0.88 (t, *J* = 6.8 Hz, 3H).
^{13}C NMR (100 MHz): δ = 153.98, 139.12, 137.43, 136.94, 135.62, 130.95, 127.22, 123.18, 121.67, 114.67, 107.62, 73.54, 69.42, 36.55, 32.63, 32.01, 30.42, 29.83, 29.78, 29.69, 29.56, 29.46, 29.29, 26.16, 22.78, 14.20.
Elemental analysis calcd (%) for $C_{47}H_{77}BF_4N_2O_3$: C, 70.13; H, 9.64; N, 3.48. Found: C, 70.15; H, 9.80; N, 3.67.
MS (MALDI-TOF): calcd. for $[M - BF_4]^+$, 717.59. Found 717.55.

1-[3,5-Bis(9,11-dodecadienyl)oxy)-4-dodecylbenzyl]pyridinium chloride (**5**(12)**-Cl**)
To a solution of **9**(12) (0.95 g, 1.41 mmol) in dry toluene (5 mL), pyridine (4 mL) was added and heated at 70 °C for 40 h. The reaction mixture was poured into water and extracted three times with $CHCl_3$. The organic phase was washed with 5% HCl aq. and sat. NaCl aq., dried over $MgSO_4$, filtered, and the solvent was removed in vacuo. The crude product was purified by flash column chromatography (silica gel, eluent: CH_2Cl_2/methanol = 10/1) and recrystallization in acetone to give **5**(12)**-Cl** (0.34 g, 45%) as white solids.
^{1}H NMR (400 MHz): δ = 9.58 (d, *J* = 6 Hz, 2 H), 8.35 (t, *J* = 7.8 Hz, H), 7.97 (d, *J* = 7.0Hz, 2H), 6.86 (s, 2 H), 6.30 (dt, *J* = 17.2, 10.4 Hz, 2H), 6.19 (s, 2H), 6.04 (dd, *J* = 15.4, 10.4 Hz, 2H), 5.70 (dt, *J* = 14.8, 7.3 Hz, 2H), 5.07 (d, *J* = 17.2 Hz, 2H), 4.94 (d, *J* = 10.0 Hz, 2H) , 3.99–3.90 (m, 9H), 2.07 (q, *J* = 7.2Hz, 4H), 1.82–1.69 (m, 6H), 1.47–1.26 (m, 38 H), 0.88 (t, *J* = 6.8 Hz, 3H).
^{13}C NMR (100 MHz): δ = 154.06, 145.12, 144.71, 137.43, 135.62, 130.95, 128.02, 127.34, 114.+7, 108.32, 73.55, 69.57, 65.02, 32.64, 32.02, 30.40, 29.83, 29.68, 29.55, , 29.45, 29.27, 26.18, 22.78, 14.21
Elemental analysis calcd (%) for $C_{48}H_{76}ClNO_3$: C, 76.81; H, 10.21; N, 1.87. Found: C, 75.77; H, 10.30; N, 1.95.
MS (MALDI-TOF): calcd. for $[M - Cl]^+$, 714.58. Found 715.02

Compound **5**(12) was synthesized analogously to **1**(12) except for using **5**(12)**-Cl** (0.25 g, 0.33 mmol) as the start compound and purified through flash column chromatography (silica

gel, eluent: CH_2Cl_2/methanol = 15/1) to give the product as white solid (0.24 g, 0.29 mmol, 88%).
^{1}H NMR (400 MHz): δ = 8.85 (d, *J* = 5.2 Hz, 2 H), 8.37 (t, *J* = 7.6 Hz, H), 7.94 (d, *J* = 7.2Hz, 2H), 6.69 (s, 2 H), 6.30 (dt, *J* = 17.1, 10.5 Hz, 2H), 6.04 (dd, *J* = 15.6, 10.4 Hz, 2H), 5.75 (dt,*J* = 15.5, 7.7 Hz, 2H), 5.65 (s, 2H), 5.07 (d, *J* = 16.8 Hz, 2H), 4.94 (d, *J* = 10.0 Hz, 2H) , 3.99-3.90 (m, 9H), 2.07 (q, *J* = 7.2Hz, 4H), 1.82–1.69 (m, 6H), 1.47–1.26 (m, 38 H), 0.88 (t, *J* = 6.8 Hz, 3H).
^{13}C NMR (100 MHz): δ = 154.16, 145.28, 144.30, 139.47, 137.43, 135.62, 130.95, 128.29, 126.65, 114.68, 108.00, 73.55, 69.40, 65.66, 32.64, 32.02, 30.42, 29.84, 29.78, 29.96, 29.56, , 29.47, 29.29, 26.18, 22.78, 14.21
Elemental analysis calcd (%) for $C_{48}H_{76}BF_4NO_3$: C, 71.89; H, 9.55; N, 1.75. Found: C, 72.07; H, 9.72; N, 1.78.
MS (MALDI-TOF): calcd. for $[M - BF_4]^+$, 714.58. Found 714.90.

2. Liquid-Crystalline Properties

DSC thermograms and XRD patterns of monomers in the LC state and their polymers are shown below. DSC thermograms and XRD patterns for **1**(12) and **1**(14) were reported in the previous papers.[1,2]

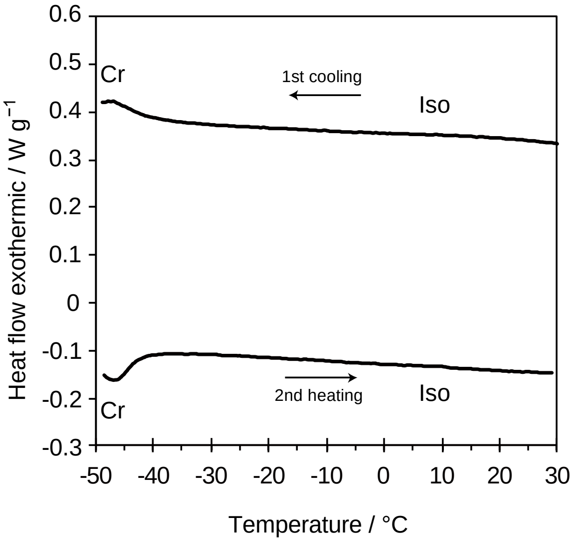

Figure S1. DSC thermograms of compound **1**(10).

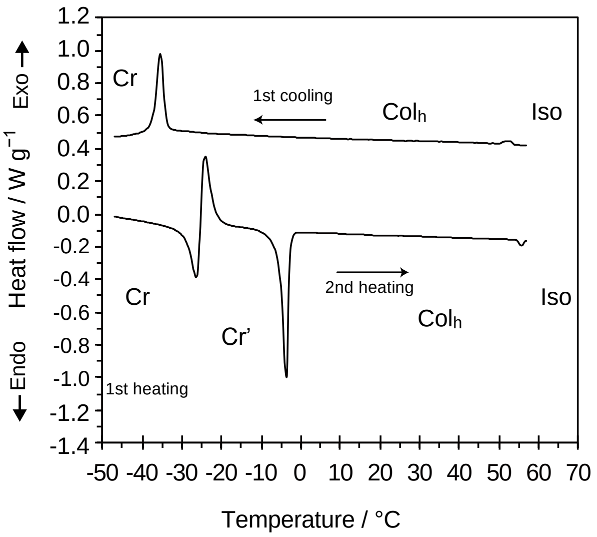

Figure S2. DSC thermograms of compound **2**(10)**.**

.

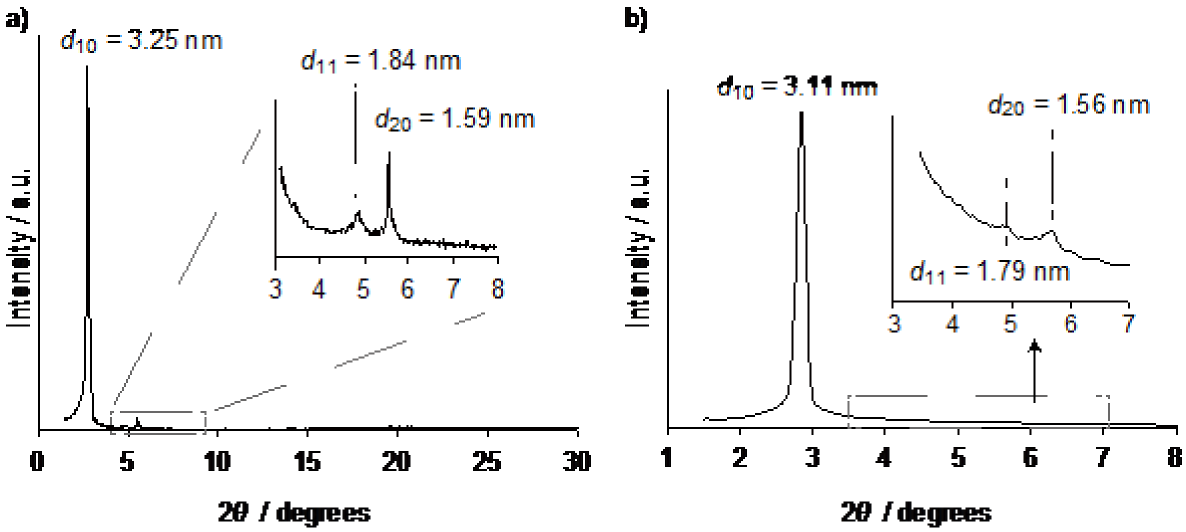

Figure S3. (a) XRD pattern of **2**(10) in the bulk film state at 25 °C and (b) SAXS pattern of the polymer film of **2**(10) after photopolymerization at 10 °C.

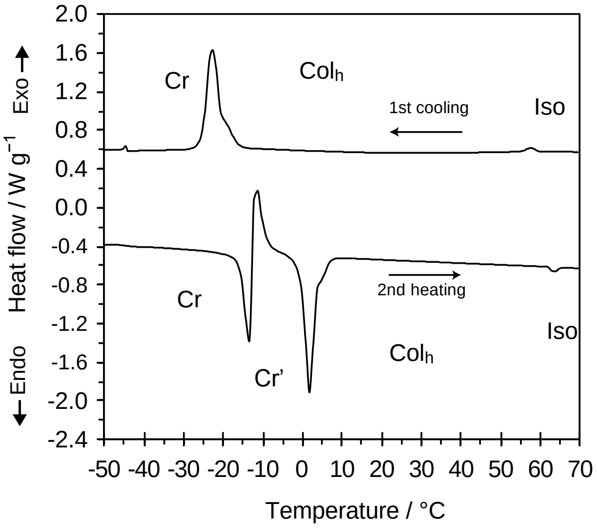

Figure S4. DSC thermograms of compound **2**(12).

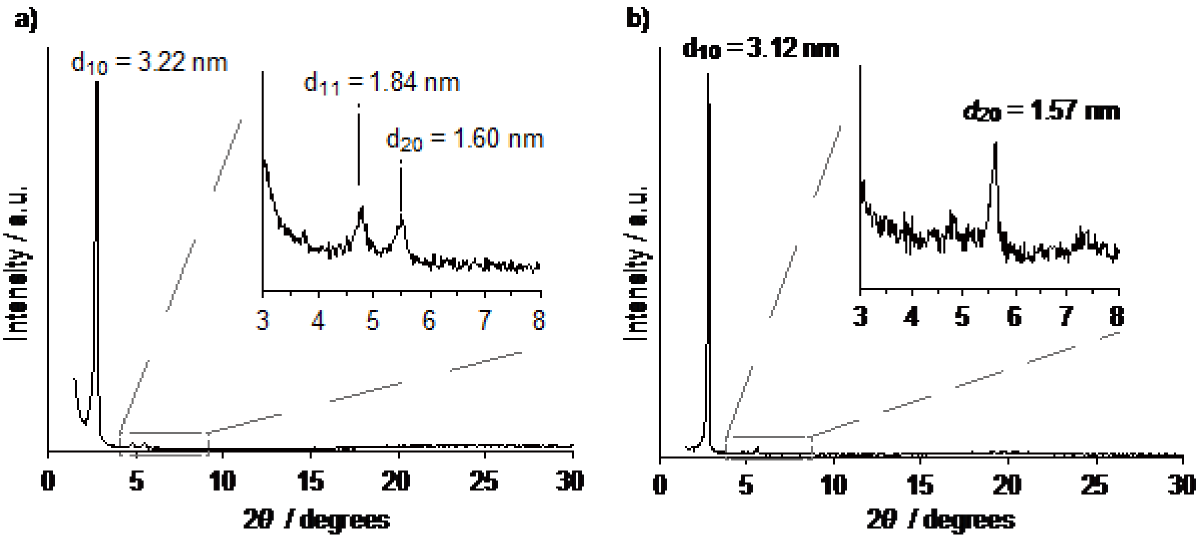

Figure S5. XRD pattern of **2**(12) in the bulk film (a) before and (b) after photopolymerization at 25 °C.

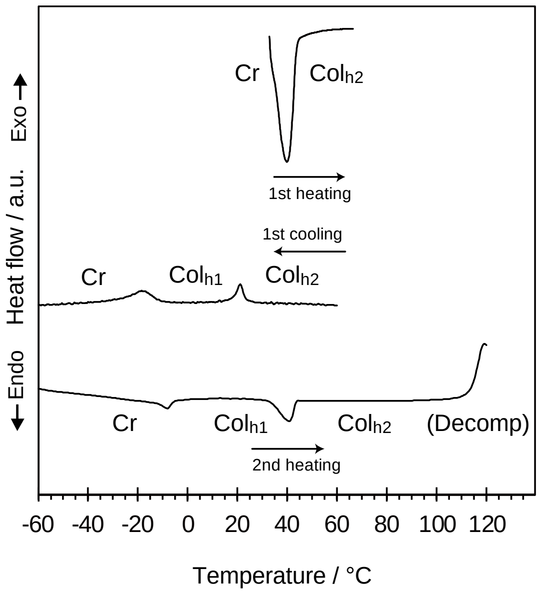

Figure S6. DSC thermograms of compound **3**(12).

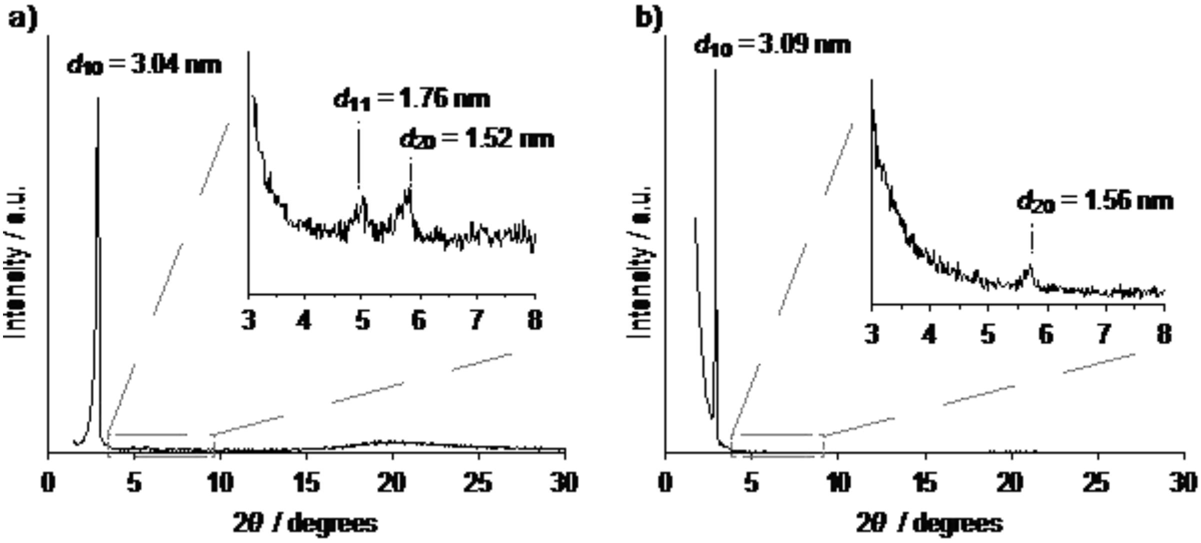

Figure S7. XRD pattern of **3**(12) in the bulk film (a) before at 60 °C and (b) after photopolymerization at 25 °C.

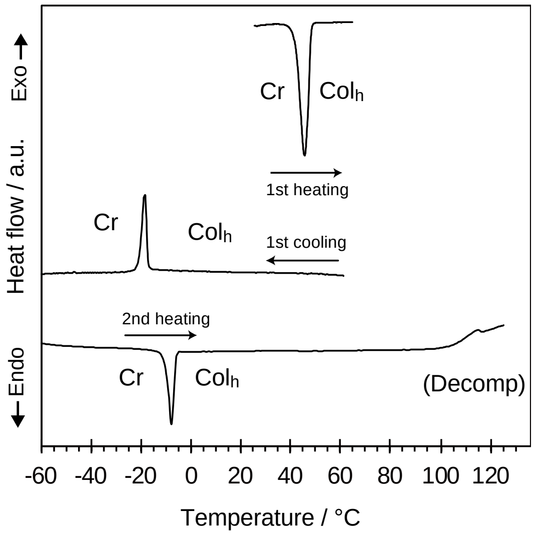

Figure S8. DSC thermograms of compound **4**(12).

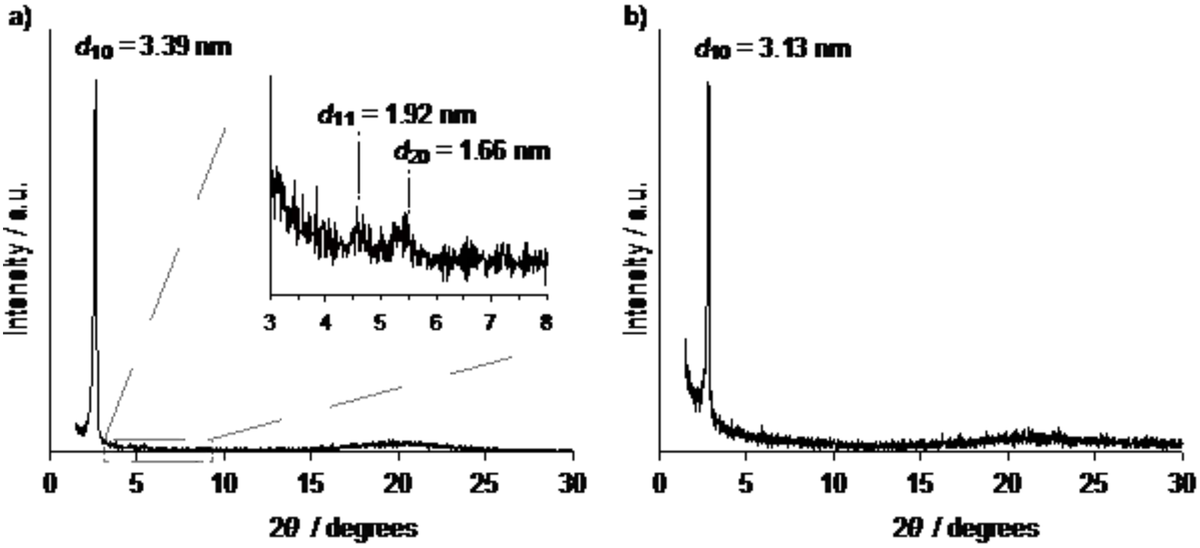

Figure S9. XRD pattern of **4**(12) in the bulk film (a) before at 50 °C and (b) after photopolymerization at 25 °C.

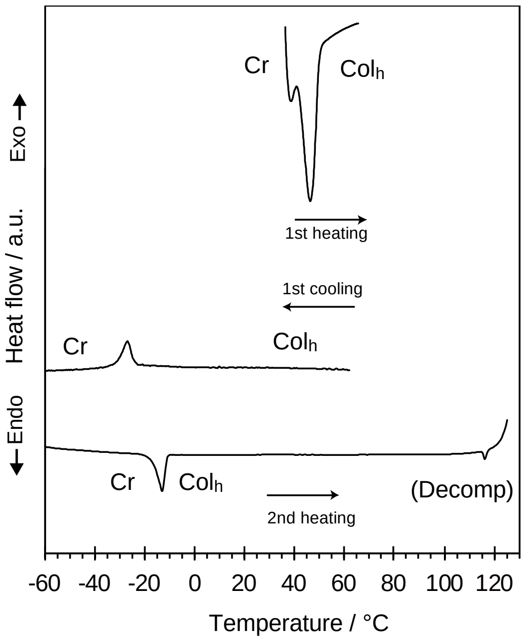

Figure S10. DSC thermograms of compound **5**(12).

.

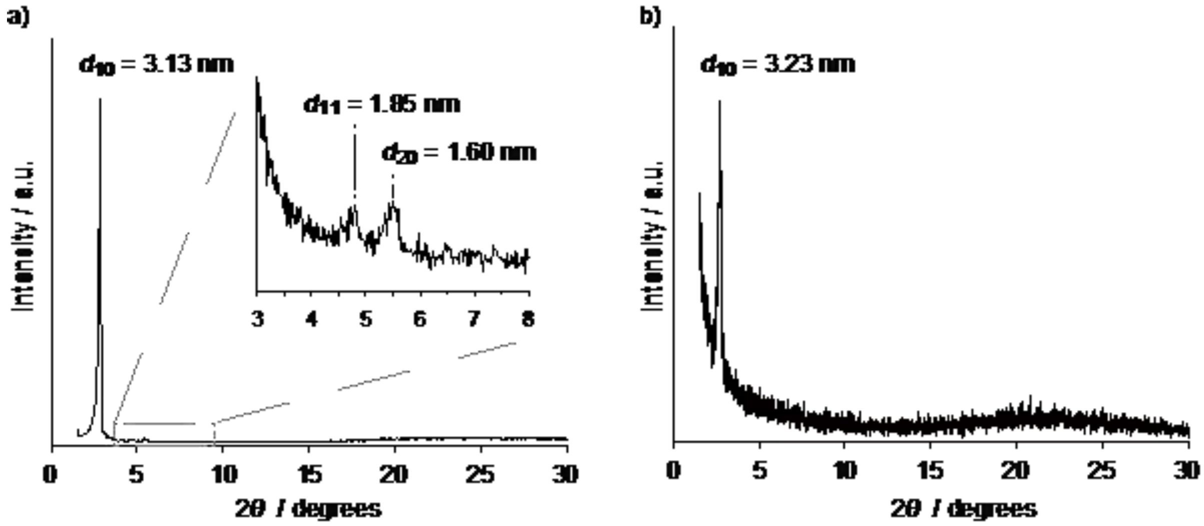

Figure S11. XRD pattern of **5**(12) in the bulk film (a) before at 60 °C and (b) after photopolymerization at 25 °C.

The lattice size and number of the molecules and channels (= sub-nanopores) in a unit area and unit volume for the LC structures are summarized in **Table S1**.

Table S1. Lattice size and number of molecules forming channels.

compound	cationic moiety	phase	lattice size (*a*) [nm]	number of molecules per 0.45-nm-thick slice of the lattice	number of the LC channels per 1.0 μm × 1.0 μm squre	total length of the LC channels within the film with H 1.0 μm × W 1.0 μm × t 0.1 μm [μm]
**1**(12)	triethylammonium	Cub_{bi} ($Ia\bar{3}d$)	8.12	23.5	6.0×10^{4} [a]	1.3×10^{4} [b]
**1**(14)	triethylammonium	Cub_{bi} ($Ia\bar{3}d$)	8.60	21.7	5.4×10^{4} [a]	1.1×10^{4} [b]
**2**(10)	diethyl-methylammonium	Col_h	3.70	4.1	8.4×10^{4}	8.4×10^{3}
**2**(12)	diethyl-methylammonium	Col_h	3.70	4.0	8.4×10^{4}	8.4×10^{3}
**3**(12)	trimethylammonium	Col_h	3.51	3.7	9.4×10^{4}	9.4×10^{3}
**4**(12)	*N*-methylimidazolium	Col_h	3.86	4.3	7.7×10^{4}	7.7×10^{3}
**5**(12)	pyridinium	Col_h	3.66	3.9	8.6×10^{4}	8.6×10^{3}

[a] calculation assuming number of the LC channels per the Cub_{bi} lattice to be 4
[b] calculation assuming total length of the LC channels with in a Cub_{bi} lattice to be $24\times a/\sqrt{8}$.[6]

Numbers of molecules per 0.45 nm thick slice were calculated with following equeation:

for the cubic structures

$$n = \frac{a^2 h\rho}{M_w / N_A}$$

for the Col_h phases

$$n = \frac{\frac{\sqrt{3}}{2}a^2 h\rho}{M_w / N_A}$$

where: a: lattice size of columnar; h: height of the slice (= 0.45 nm); ρ: density of the LC film (1 g cm^{-3}); M_w: molecular weight (g mol^{-1}); N_A: Avogadro constant (mol^{-1})

3. Simulation for the Ionic Channels
3-1. Potential Parameters

Three LC monomers with three kinds of ammonium moieties (LC monomer with a triethylammonium moiety (= **1**(*n*)), that with a methyl-diethylammonium moiety (= **2**(*n*)), and that with a trimethylammonium moiety (= **3**(*n*)) were examined.

The interaction acting on the LC monomer was estimated as the sum of intermolecular potentials (the Coulomb and the Lennard-Jones (LJ) potentials) for all atoms of the monomer plus the sum of intramolecular potentials (bond, valent angle, and dihedral angle potentials). Parameters for the LJ on each atom, bond, valent angle, and dihedral angle potentials were determined with a general Amber force field (GAFF).[8] The charge (q) on each atom was generated with ANTECHAMBER 1.4.[9] The potential parameters in the GAFF are optimized with the TIP3P model.[10] Therefore, the TIP3P model was used for estimation of the intermolecular interaction between a pair of water molecules. The interaction acting on each of Na^+ and Cl^- ions was estimated with a model proposed by Joung et al.[11]

The LJ parameters, ε and σ, for the interactions between the LC monomer and water molecule, and between the LC monomer and each of the ions (the LC–water and LC–ion interactions, respectively) were determined by the Lorentz-Berthelot rules. We checked that for more than 20 different LC–water distances, the potential energy, U, for the LC–water interaction in the present potential models was satisfactorily reproduced by the first-principles calculation using the MP2 method with the basis function of 6-311+G**. However, U for the LC–ion interaction in the present potential models was deviated from that calculated by the first-principles calculation. Thus, to reduce the deviation of U, σ for the LC–ion interaction, which was determined by the Lorentz-Berthelot rules, was multiplied by 0.9. The reason for use of the MP2 method to evaluate U was that the method has the advantage of naturally and properly accounting for medium- and long-range correlation effects, compared to other methods, such as the density functional theory method.[12]]

This study focused on the structure of an ionic channel enclosed by the arrangement of ammonium moieties of the LC monomers, and the mobility of water molecules and ions in the channel. Thus, for simplicity, MD simulations in this study were performed with a model of the LC monomer from which alkyl chains were eliminated (hereafter, LC monomer model). The potential parameters for each atomic site of the benzene ring and ammonium moiety of the LC monomer model are listed in **Table S2**, and the definitions of atomic sites are given in **Figure S12**. q, and ε and σ of the Lennard-Jones (LJ) potential, $U_{LJ} = 4\varepsilon\{(\sigma/r)^{12} - (\sigma/r)^6\}$ (r is the distance between a pair of sites), on the atomic sites of each LC monomer model. q on

each atomic site was determined from the first-principles calculation for all atoms of the LC monomer using ANTECHAMBER. The whole LC monomer has a charge of +1. However, the sum of q over the atoms of the LC monomer model was not equal to +1. In this study, the charge of the LC monomer model was adjusted to +1 with q on C3.

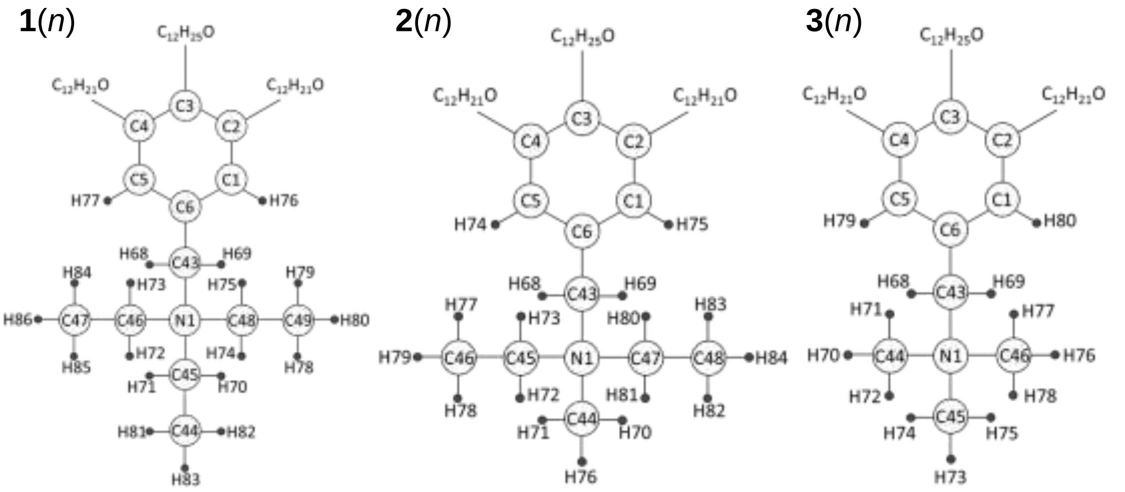

Figure S12. The definitions of the atomic sites in the LC monomer models.

Table S2. The charge (q) and potential parameters (ε, σ) for each atomic site of the benzene ring and ammonium moiety of the LC monomer model.

**1**(*n*)	C1	C2	C3	C4	C5	C6	N11
q [eC]	-0.293297	0.063165	-0.004115	0.260638	-0.293297	0.064509	-0.152894
ε [kJ/mol]	0.3598	0.3598	0.3598	0.3598	0.3598	0.3598	0.7113
σ [nm]	0.3400	0.3400	0.3400	0.3400	0.3400	0.3400	0.3250
	C43	H68	H69	C44	C45	H70	H71
q [eC]	-0.194313	0.151863	0.151863	0.110138	0.110138	0.060731	0.060731
ε [kJ/mol]	0.4577	0.0657	0.0657	0.4577	0.4577	0.0657	0.0657
σ [nm]	0.3400	0.1960	0.1960	0.3400	0.3400	0.1960	0.1960
	H72	C46	C47	H73	H74	C48	H75
q [eC]	0.060731	-0.177088	-0.177088	0.060731	0.187759	0.110138	0.187759
ε [kJ/mol]	0.0657	0.4577	0.4577	0.0657	0.0657	0.4577	0.0657
σ [nm]	0.1960	0.3400	0.3400	0.1960	0.1960	0.3400	0.1960
	C49	H76	H77	H78	H79	H80	H81
q [eC]	-0.177088	0.078536	0.078536	0.078536	0.078536	0.078536	0.078536
ε [kJ/mol]	0.4577	0.0628	0.0628	0.0657	0.0657	0.0657	0.0657
σ [nm]	0.3400	0.2600	0.2600	0.2650	0.2650	0.2650	0.2650
	H82	H83	H84	H85	H86		
q [eC]	0.060731	0.060731	0.078536	0.078536	0.078536		
ε [kJ/mol]	0.0657	0.0657	0.0657	0.0657	0.0657		
σ [nm]	0.2650	0.2650	0.2650	0.2650	0.2650		

**2**(*n*)	C1	C2	C3	C4	C5	C6	N1
q [*e*C]	-0.229175	0.226162	-0.183183	0.226162	-0.229175	-0.010191	-0.01443
ε [kJ/mol]	0.3598	0.3598	0.3598	0.3598	0.3598	0.3598	0.7113
σ [nm]	0.3400	0.3400	0.3400	0.3400	0.3400	0.3400	0.3250
	C43	H68	H69	C44	C45	H70	H71
q [*e*C]	-0.176513	0.14788	0.14788	-0.238343	0.118724	0.138846	0.138846
ε [kJ/mol]	0.4577	0.0657	0.0657	0.4577	0.4577	0.0657	0.0657
σ [nm]	0.3400	0.1960	0.1960	0.3400	0.3400	0.1960	0.1960
	H72	C46	C47	H73	H74	C48	H75
q [*e*C]	0.056497	-0.20281	0.118724	0.056497	0.173801	-0.20281	0.173801
ε [kJ/mol]	0.0657	0.4577	0.4577	0.0657	0.0628	0.4577	0.0628
σ [nm]	0.1960	0.3400	0.3400	0.1960	0.2600	0.3400	0.2600
	H76	H77	H78	H79	H80	H81	H82
q [*e*C]	0.138846	0.085159	0.085159	0.085159	0.056497	0.056497	0.085159
ε [kJ/mol]	0.0657	0.0657	0.0657	0.0657	0.0657	0.0657	0.0657
σ [nm]	0.1960	0.2650	0.2650	0.2650	0.1960	0.1960	0.2650
	H83	H84					
q [*e*C]	0.085159	0.085159					
ε [kJ/mol]	0.0657	0.0657					
σ [nm]	0.2650	0.2650					

**3**(*n*)	C1	C2	C3	C4	C5	C6	C43
q [*e*C]	-0.308209	0.277377	-0.217685	0.277377	-0.308209	0.053544	-0.094756
ε [kJ/mol]	0.3598	0.3598	0.3598	0.3598	0.3598	0.3598	0.4577
σ [nm]	0.3400	0.3400	0.3400	0.3400	0.3400	0.3400	0.3400
	C44	C45	C46	N1	H68	H69	H70
q [*e*C]	-0.327552	-0.327552	-0.327552	0.134496	0.127527	0.127527	0.171085
ε [kJ/mol]	0.4577	0.4577	0.4577	0.7113	0.0657	0.0657	0.0657
σ [nm]	0.3400	0.3400	0.3400	0.3250	0.1960	0.1960	0.1960
	H71	H72	H73	H74	H75	H76	H77
q [*e*C]	0.171085	0.171085	0.171085	0.171085	0.171085	0.171085	0.171085
ε [kJ/mol]	0.0657	0.0657	0.0657	0.0657	0.0657	0.0657	0.0657
σ [nm]	0.1960	0.1960	0.1960	0.1960	0.1960	0.1960	0.1960
	H78	H79	H80				
q [*e*C]	0.171085	0.186951	0.186951				
ε [kJ/mol]	0.0657	0.0628	0.0628				
σ [nm]	0.1960	0.2600	0.2600				

We note that it would be better to use more accurate potential parameters, which were determined so that they matched solution free energies, especially for the LJ interactions, if the purpose of the simulation was quantitative reproduction of the structural and dynamic properties of ions in channels created by real self-assembled LC monomers. However, the purpose of the simulation was qualitative understanding of the difference in the mobility of water molecules and ions in the channel between **1(*n*)**, **2(*n*)**, and **3(*n*)**. Moreover, we checked

that for both the LC–water and LC–ion interactions during the simulation, *U* for the Coulomb interaction was much greater than that for the LJ interaction, suggesting that the LJ interactions did not significantly influence the mobility of water molecules and the ions in the channel. Thus, we believe that the present potential parameters were sufficient for this study.

3-2. Simulation Systems

Two simulation systems were prepared: one was the system used for an MD simulation to investigate the stable structure of the channel created by the LC monomers (system A), and the other was the system used for an MD simulation to investigate the mobility of water molecules, Na^+ and Cl^- ions in the channel (system B).

System A was created in the following way (see **Figure S13**). Firstly, an LC tetramer layer was formed with four LC monomer models (Figure S13a). In the layer, the LC monomer models were arranged radially on positions rotated by 90° so that their ammonium moieties were oriented toward the center of the layer. Secondly, an assembly of four LC tetramer layers was constructed to create an ionic channel at the center of it by piling up the layers in the *z*-axis direction (Figure S13b). In the assembly, the second and forth layers were rotated by 45° around the center to form a close-packed structure. The distance between the layers was fixed at 0.45 nm. To make the assembly electrically neutral, 16 Cl^- ions were inserted into the channel of the assembly. Notably, the experimental study used BF_4^- ions but not Cl^- ions. However, we checked that the minimum energy structure of the assembly with Cl^- ions did not significantly change even if SO_4^{2-} ions were used instead. Therefore, we assumed that the minimum energy structure with Cl^- ions was the same as that with BF_4^- ions. Again, the purpose of the simulation was qualitative understanding of the difference in the mobility of water molecules and ions in the channel between **1(*n*)**, **2(*n*)**, and **3(*n*)**, but not quantitative reproduction of the structural and dynamic properties of ions in real channels created by the self-organized LC monomers. Then, system A was constructed by putting the assembly into a rectangular-parallelepiped (Figure S13c). The dimension of system A in the *z* direction, 1.8 nm, was set to be equal to the length of the assembly in the *z* direction. The dimensions in the *x* and *y* directions were 3×3 nm^2. Periodic boundary conditions were imposed in all *x*, *y* and *z* directions.

In this study, several different sizes of the channel in the assembly were examined. The size of the channel was changed by changing the distance between the centers of the LC tetramer layer and benzene ring, *R*.

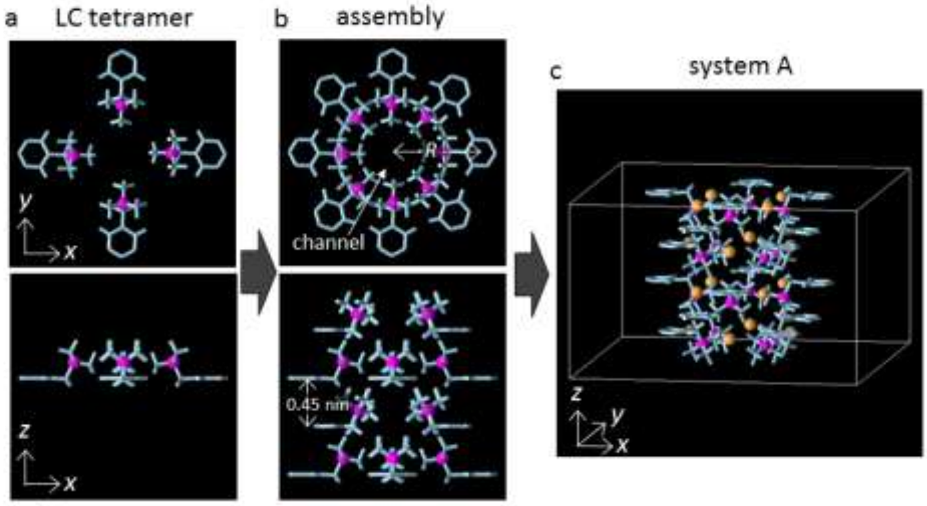

Figure S13. Schematic of the procedure to create system A for **3**(*n*).

System B was created in the following way. Firstly, a grand canonical MC (GCMC) simulation was performed for system A to fill the system with water molecules. In the GCMC simulation, temperature was set to 298 K and the chemical potential was set to the value for the bulk water at 298 K and 10 atm in the TIP3P model (−26 kJ/mol). The total number of trials (translation, rotation, insertion and removal of water molecules) in the GCMC simulation was 1.4×10^{10}. Then, using the final configuration generated by the GCMC simulation, system B was created by putting a copy of the system filled with water molecules onto the original one in the *z*-axis direction.

Because we focused on the mobility of water molecules and ions in the channel, water molecules and ions located at positions far from the channel were eliminated from the system. A water molecule was judged to be located at a position far from the channel if it was not included in a circular column with a radius of *R* nm from the center of the channel. Two water molecules existing in the channel were replaced with single Na^+ and Cl^- ions. Thus, system B consisted of eight LC tetramer layers, a Na^+ ion, 17 Cl^- ions (16 counter anions of the assembly and the anion dissolved in water), and water molecules. The dimensions of system B were $5\times5\times3.6$ nm^3. Periodic boundary conditions were imposed in all *x, y* and *z* directions.

3-3. MD Simulation and Analysis

Computation was carried out using a leap-frog algorithm with a time step of 0.5 fs. Temperature was maintained at a constant value by means of a Nosé-Hoover thermostat with

a coupling parameter of 0.1 ps.[13] The O–H distances in the LC monomer were kept constant at their equilibrium values by means of the SHAKE algorithm. The long-range Coulomb interactions were estimated using the Ewald summation method. The real space cut-off distance was set to 1 nm. The Ewald convergence parameter was set to 4.658 nm^{-1} for system A and 3.208 $nm^{-1}$for system B. The maximum indices of the reciprocal lattice vector in the *x*, *y*, and *z*-directions were 14, 14, and 8 for system A and 16, 16, and 12 for system B. The LJ interactions were cut-off at an interatomic distance of 1 nm. The simulations were performed with DL_POLY 2.20.[14] The distance restraints and angler restraints implemented in DL_POLY 2.20 were used to maintain the bond lengths and angles of the LC monomer the around their equilibrium values.

For system A, MD simulations were performed to obtain the energetically stable structure of the channel in the following way. First, an MD simulation was performed at 298 K for 1.5 ns. Then, using the final configuration of the MD simulation at 298 K, an MD simulation was performed at 0 K for 0.5 ns. These heating and subsequent quenching simulations were repeated three times, starting with different initial configurations. The energetically stable structure of the channel was determined by comparing the potential energy of the final configuration for three quenching simulations.

The stable structure of the channel for each R was analyzed with the final configuration of the simulation. For system B, three MD simulations starting with various initial positions of Na^+ and Cl^- ions were performed at 298 K. The run for each simulation was 15 ns. The mobility of water molecules and ions in the channel was investigated by analyzing the root mean square displacement, $\langle dr^2 \rangle$. For each of the three simulations, the analysis was done using the time-sequence of the coordinates of water molecules and ions at eight different periods. Thus, for each of water molecules, Na^+ and Cl^- ions, the $\langle dr^2 \rangle$ as a function of t was created by averaging 24 independent $\langle dr^2 \rangle$ functions. Notably, during the simulation, 16 Cl^- ions as the counter anions of the assembly and a Cl^- ion as the anion dissolved in water were indistinguishable. Thus, the $\langle dr^2 \rangle$ function for Cl^- ion was created with the $\langle dr^2 \rangle$ data for all the 17 Cl^- ions.

During the simulation for both systems, the benzene rings of the LC monomer models were fixed at their initial positions. During the simulation for system B, an external potential, $U_{\mathrm{ext}} = A(R-r_{xy})^{-n}$ (r_{xy} is the distance between the center of the system and species in the x–y plane) was applied to the system in order to prevent water molecules and ions from moving out of the channel. A and n used in this study were 0.001 kJ/mol and 4, respectively.

3-4. MD Simulations for Different Pore Sizes

To check the effect of the space volume size on the mobility of water molecules and the ions in the nanopore, an MD simulation to investigate the mobility of them was also performed for the nanopore of **3**(*n*) with two different sizes, R = 0.8 and 0.875 nm. The simulations results of $\langle dr^2 \rangle$ as a function of t for all R are shown in **Figure S14**. The self-diffusion coefficient of water molecules increased with increasing R, whereas the coefficients of the ions did not significantly change with R. This result suggests that the lower mobility of water molecules in the nanopore for **3**(*n*) than for **1**(*n*) and **2**(*n*) originated from the smaller size of the space volume.

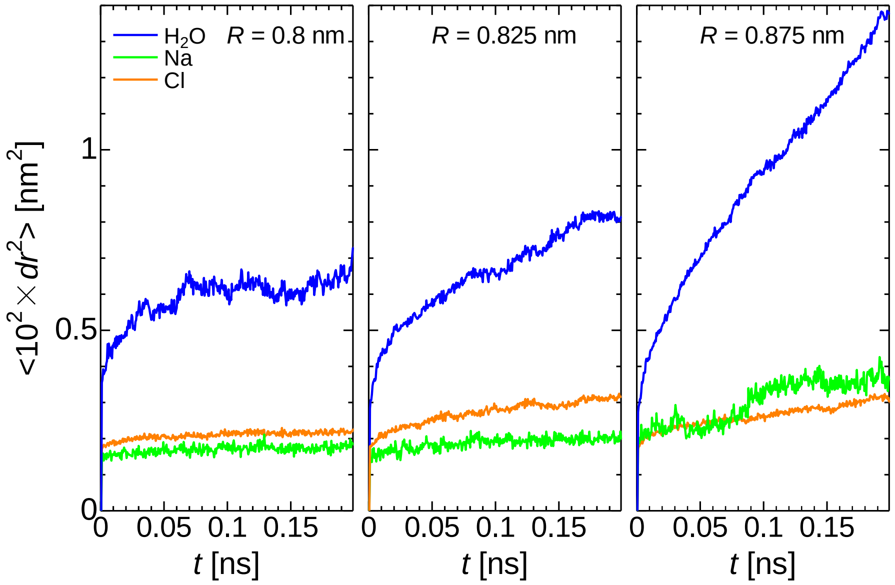

Figure S14. The $\langle dr^2 \rangle$ as a function of t for water molecules, Na^+ ion, and Cl^- ion for **3**(*n*) with R = 0.8, 0.825, and 0.875 nm

4. References for Supporting Information

[1] T. Ichikawa, M. Yoshio, A. Hamasaki, J. Kagimoto, H. Ohno, T. Kato, *J. Am. Chem. Soc.* **2011**, *133*, 2163–2169.

[2] N. Marets, D. Kuo, J. Torrey, T. Sakamoto, M. Henmi, H. Katayama, T. Kato, *Adv. Healthcare Mater.* **2017**, *6*, 1700252.

[3] T. Nishiguchi, S. Hayakawa, Y. Hirasaka, M. Saitoh, *Tetrahedron Lett.* **2000**, *41*, 9843–9846.

[4] T. Asawa, Y. Sugiyama, M. Kirihara, T. Iwai, Y. Kimura, *Synlett* **2014**, *25*, 0596–0598.

[5] Y. Wang, A. M. Arif, F. G. West, *J. Am. Chem. Soc*. **1999**, *121*, 876–877.

[6] S. Kutsumizu, H. Mori, M. Fukatami, S. Naito, K. Sakajiri, K. Saito, *Chem. Mater.* **2008,** *20*, 3675–3687.

[7] M. Henmi, K. Nakatsuji, T. Ichikawa, H. Tomioka, T. Sakamoto, M. Yoshio, T. Kato, *Adv. Mater*. **2012**, *24*, 2238–2241.

[8] J. Wang, R. M. Wolf, J. W. Caldwell, P. A. Kollman, D. A. Case, *J. Compt. Chem.* **2004**, *25*, 1157–1174.

[9] J. Wang, W. Wang, P. A. Kollman, D. A. Case, *J. Mol. Graph.* **2006**, *25*, 247–260.

[10] W. L. Jorgensen, J. Chandrasekhar, J. D. Madura, R. W. Impey, M. L. Klein, *J. Chem. Phys.* **1983**, *79*, 926–932.

[11] I. S. Joung, T. E. Cheatham, III, *J. Phys. Chem. B* **2008**, *112*, 9020–9041.

[12] J. Deng, A. T. B. Gilbert, P. M. W. Gill, *J. Chem. Thoer. Compt.* **2015**, *11*, 1639–1644.

[13] W. G. Hoover, *Phys. Rev. A* **1985**, *31*, 1695–1697.

[14] W. Smith, T. R.Forester, *J. Mol. Graphics* **1996**, *14*, 136–141.
